# Identification of Medicare Recipients at Highest Risk for *Clostridium difficile* Infection in the US by Population Attributable Risk Analysis

**DOI:** 10.1371/journal.pone.0146822

**Published:** 2016-02-09

**Authors:** Erik R. Dubberke, Margaret A. Olsen, Dustin Stwalley, Ciarán P. Kelly, Dale N. Gerding, Yinong Young-Xu, Cedric Mahé

**Affiliations:** 1 Department of Medicine, Washington University School of Medicine, St. Louis, Missouri, United States of America; 2 Department of Surgery, Washington University School of Medicine, St. Louis, Missouri, United States of America; 3 Division of Gastroenterology, Beth Israel Deaconess Medical Center, Harvard Medical School, Boston, Massachusetts, United States of America; 4 Edward Hines Jr. Veterans Affairs Hospital, Hines, Illinois, and Loyola University Chicago Stritch School of Medicine, Maywood, Illinois, United States of America; 5 Department of Psychiatry, Geisel School of Medicine at Dartmouth, Hanover, New Hampshire, United States of America; 6 Sanofi-Pasteur, Lyon, France; Cleveland Clinic, UNITED STATES

## Abstract

**Background:**

Population attributable risk percent (PAR%) is an epidemiological tool that provides an estimate of the percent reduction in total disease burden if that disease could be entirely eliminated among a subpopulation. As such, PAR% is used to efficiently target prevention interventions. Due to significant limitations in current *Clostridium difficile* Infection (CDI) prevention practices and the development of new approaches to prevent CDI, such as vaccination, we determined the PAR% for CDI in various subpopulations in the Medicare 5% random sample.

**Methods:**

This was a retrospective cohort study using the 2009 Medicare 5% random sample. Comorbidities, infections, and healthcare exposures during the 12 months prior to CDI were identified. CDI incidence and PAR% were calculated for each condition/exposure. Easy to identify subpopulations that could be targeted from prevention interventions were identified based on PAR%.

**Findings:**

There were 1,465,927 Medicare beneficiaries with 9,401 CDI cases for an incidence of 677/100,000 persons. Subpopulations representing less than 15% of the entire population and with a PAR% ≥ 30% were identified. These included deficiency anemia (PAR% = 37.9%), congestive heart failure (PAR% = 30.2%), fluid and electrolyte disorders (PAR% = 29.6%), urinary tract infections (PAR% = 40.5%), pneumonia (PAR% = 35.2%), emergent hospitalization (PAR% = 48.5%) and invasive procedures (PAR% = 38.9%). Stratification by age and hospital exposures indicates hospital exposures are more strongly associated with CDI than age.

**Significance:**

Small and identifiable subpopulations that account for relatively large proportions of CDI cases in the elderly were identified. These data can be used to target specific subpopulations for CDI prevention interventions.

## Introduction

The incidence and severity of *Clostridium difficile* infection (CDI) have increased significantly in the last decade, and it was the most common pathogen causing healthcare-associated infections in 2011 [1–3]. It is estimated there were 453,000 incident CDI episodes and 29,300 deaths in the US due to CDI in 2011 [4]. CDI is estimated to result in at least $4.8 billion in excess U.S. inpatient hospitalization costs [5]. In addition, CDI adds to morbidity, increasing the likelihood of discharge to long term care facilities (vs. home) by 60% and hospital readmission more than two-fold [6].

Despite increases in CDI burden, our understanding of CDI epidemiology from a population perspective remains incomplete. Most studies have focused on the hospital setting and identifying individual risk factors for CDI [7,8]. However, a population-based approach is typically used to improve health and prevent disease within a population. One tool used to identify, and target, populations at risk for a preventable disease is population attributable risk percent (PAR%) [9]. PAR% provides an estimate of the percent reduction in total disease burden (e.g. lung cancer) if the incidence of that disease among a subpopulation (e.g. smokers) could be entirely eliminated. A more comprehensive understanding of CDI epidemiology that defines both populations at higher risk and the size of those populations is needed to assist clinicians, researchers, and policy makers to develop the most effective methods for CDI surveillance and prevention, and focus these efforts on persons most likely to benefit.

Several initiatives to improve our knowledge of CDI epidemiology exist, but none address the prevalence of exposures from a population perspective needed to optimize CDI prevention. The Centers for Disease Control and Prevention (CDC) Emerging Infections Program (EIP) initiated population-based surveillance for CDI in 2009, providing important information on persons who develop CDI, the settings where CDI onset occurs, and prevalent strains in the community and hospitals [4]. However, since data are obtained only from CDI cases, it is not possible to determine the incidence of CDI for different subpopulations [10]. The Healthcare Cost and Utilization Project (HCUP) Nationwide Inpatient Sample (NIS) has been used to calculate national estimates of CDI hospitalizations. The NIS does not link patients stays over time, resulting in an over-estimation of CDI incidence and prevalence [1,11]. The CareFusion and Premier Perspective clinical research databases have also been used to study CDI [12,13]. With these datasets only inpatient data are available from a restricted subset of hospitals, limiting their usefulness to determine population characteristics associated with CDI. Private insurance claims databases have also been analyzed. Limitations of these studies include a restricted geographic area based on the insurance provider coverage and a younger, healthier patient population at lower risk of CDI [14,15].

Most of these aforementioned approaches have been focused on identifying and quantifying risk factors for CDI, but the burden of CDI in different subpopulations and the proportion of CDI cases associated with the different subpopulations have not been clearly established. This information is needed to optimize surveillance efforts, identify new opportunities for prevention, and to prioritize efficient population-based interventions. Comprehensive data are available from both the inpatient and outpatient settings in the Medicare 5% random sample, which allows for the study of CDI at the population level. To improve our understanding of CDI in the elderly from a population perspective, we conducted a retrospective cohort study using the Medicare 5% random sample.

## Methods

### Data Sources and Study Design

The 2009 Medicare 5% random sample (Chronic Condition Warehouse) was used for this study. Data on inpatient, outpatient, skilled nursing, and Part D outpatient drug data (see SI File for details) were used. Medicare Part A and Part B fee-for-service coverage from January 1, 2008 through December 31, 2009 (or through death) was required. Approval to conduct this research was obtained from the Washington University School of Medicine Human Research Protection Office. A waiver of informed consent was granted. Data were deidentified.

### Outcomes and Definitions

#### CDI case identification

The first episode of CDI was identified for each person in 2009. Criteria for CDI included any of the following:

1. International Classification of Diseases, Ninth Revision, Clinical Modification (ICD-9-CM) diagnosis code for CDI (008.45) during an inpatient hospital stay;
2. 008.45 ICD-9-CM code in an outpatient encounter (excluding claims with place of service code, provider type code, or type service code of diagnostic laboratory, or if the only UB-92 revenue center codes were for laboratory services (0300–0309));
3. Outpatient CDI therapy (non-topical metronidazole, oral vancomycin) within 14 days of a claim for a *C*. *difficile* test.

The date of CDI onset was defined as the first date with a coded diagnosis of CDI, unless additional information was available for an earlier onset based on symptoms or other indications of CDI (e.g. diagnosis of diarrhea, see S1 File). Every person was assigned an index date. For persons with CDI, the index date was the date of onset of CDI. For persons without CDI, an index date was randomly assigned such that the distribution of index dates among persons without CDI mirrored the distribution of index dates of people with CDI. Definitions for comorbidities, prior infections, and healthcare exposures are provided in S1 File.

### Analyses

The incidence of CDI per 100,000 persons was calculated overall and for all of the comorbidities, infections and healthcare exposures (the subpopulations or exposures). The relative risk and PAR% for each subpopulation were calculated using formula two in Rockhill et al[16] as follows: PAR% = (P(E) x (RR—1))/ ((P(E) x (RR—1)) + 1)

Where P(E) = prevalence of the exposure and RR = relative risk. PAR% is a comparison of the incidence rate of the outcome (i.e. CDI) in the exposed group (i.e. subpopulation: comorbidity, infection, or healthcare exposure) with the incidence in the counterfactual situation in which the exposure is absent, and gives an estimate of the percent reduction in CDI cases in the total population if the incidence of CDI among people with the exposure could be entirely eliminated [17]. CDI incidence and PAR% for each subpopulation were then stratified by time from the last exposure to the index date (≤3, 3–6, and 6–12 months, for infection and healthcare exposures only), age (66–70, 71–75, 76–80, 81–85, >85), and hospitalization in the three months prior to the index date (no hospitalization, non-emergent hospitalization, emergent hospitalization). SAS version 9.3 (Cary, N.C.) and SPSS version 21 (IBM Statistics, Chicago, IL.) were used for analyses. This study received approval from the Washington University Human Research Protection Office.

## Results

**Fig 1:**
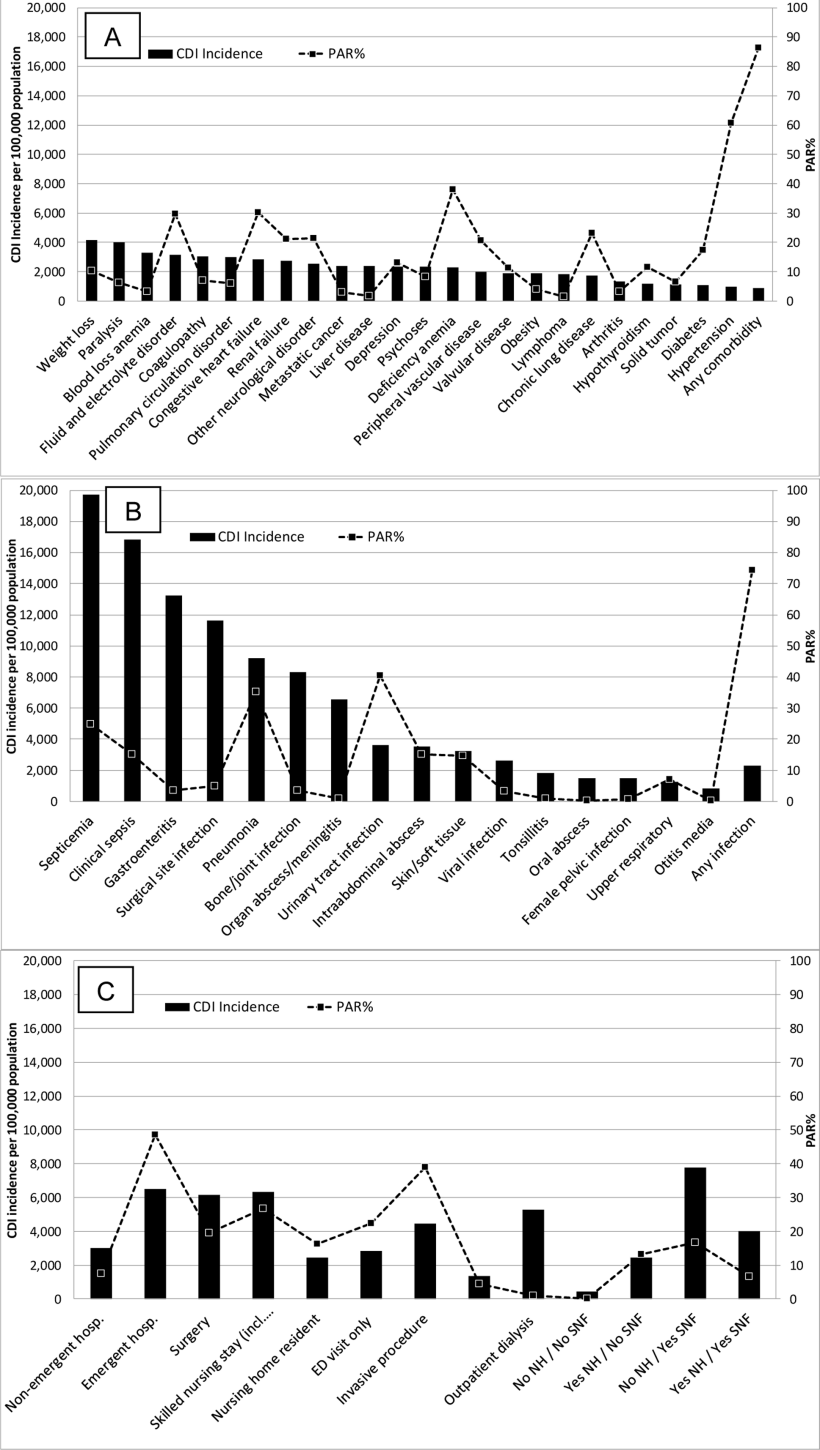
CDI incidence and CDI population attributable risk percent (PAR%) of comorbidities (A), infections within 3 months of CDI (B), and healthcare exposures within 3 months of CDI (C). No NH / No SNF = people without a nursing home or skilled nursing facility exposure within 3 months of CDI; Yes NH / No SNF = nursing home residents without a skilled nursing facility exposure within 3 months of CDI; No NH / Yes SNF = people without a nursing home exposure but with a skilled nursing facility exposure within 3 months of CDI; Yes NH / Yes SNF = nursing home residents.

**Fig 2:**
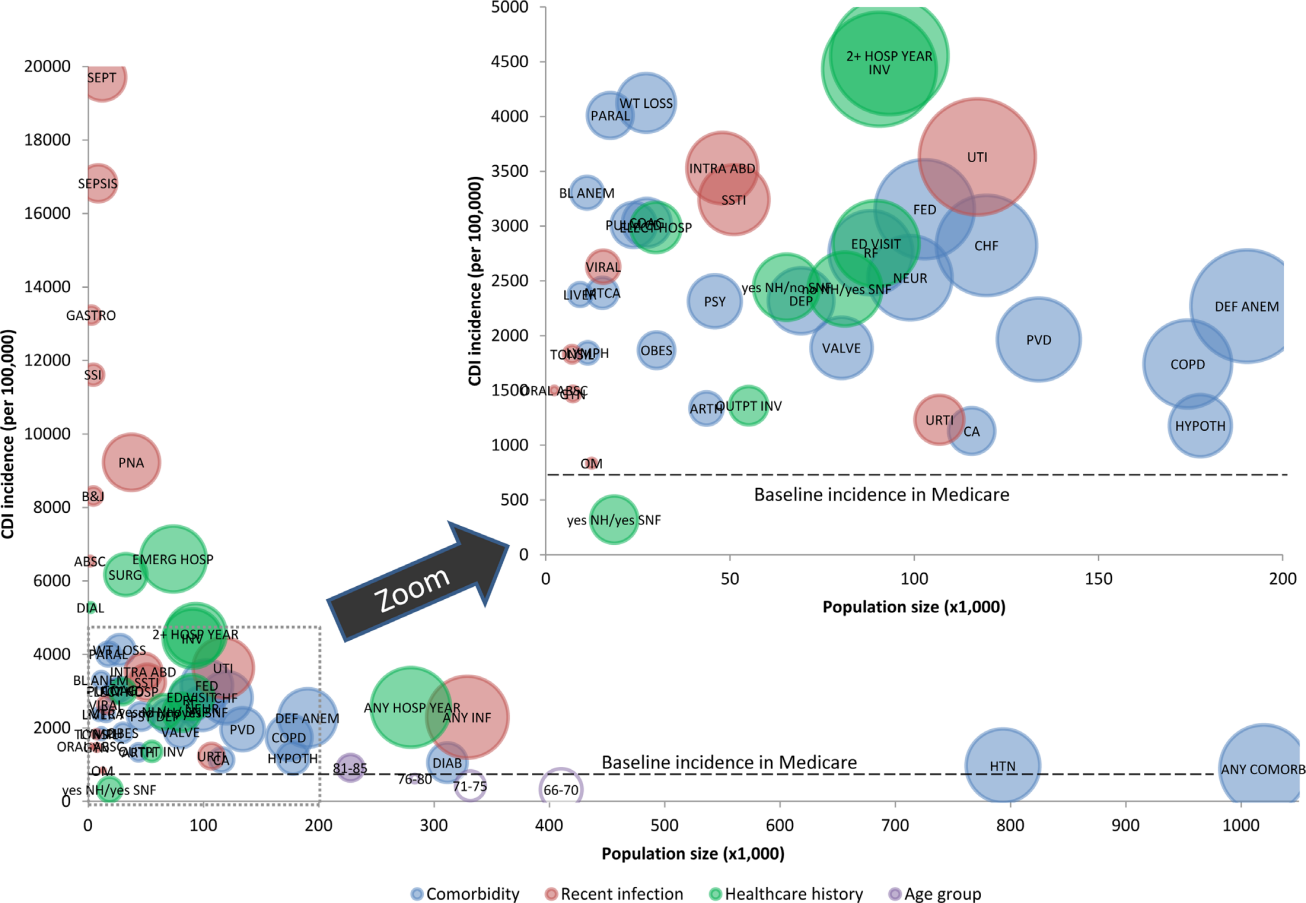
Graph of populations size (X-axis) versus CDI incidence (Y-axis) of comorbidities, infections within three months of CDI, and healthcare exposures within three months of CDI. The size of the circles represents the relative difference in population attributable risk percent (PAR%) for each exposure examined. (**Comorbidities** (blue bubbles): ANY COMORB = any comorbiditiy, ARTH = arthritis, BL ANEM = blood loss anemia, CA = solid tumor, CHF = congestive heart failure, COAG = coagulopathy, COPD = chronic lung disease, DEF ANEM = deficiency anemia, DEP = depression, DIAB = diabetes mellitus, FED = fluid and electrolyte disorder, HTN = hypertension, HYPOT4 = hypothyroid, LYMPH = lymphoma, MTCA = metastatic cancer, NEUR = other neurological disorder, OBES = obesity, PARAL = paralysis, PSY = psychoses, PULM CD = pulmonary circulatory disorder, PVD = peripheral vascular disease, RF = renal failure, VALVE = valvular disease, WT LOSS = weight loss; **Infections** (red bubbles): ABSC = abscess in organ/meningitis; ANY INF = any infection, B&J = bone/joint infection, GASTRO = gastroenteritis, GYN = female pelvic infection, INTRA ABD = intra-abdomional abscess/peritonitis, OM = otitis media, ORAL ABSC = oral abscess, PNA = pneumonia, SEPSIS = clinical sepsis, SEPT = septicemia/endovascular infection, SSI = surgical site infection, SSTI = skin/soft tissue infection, TONSIL = tonsillitis, URTI = upper respiratory tract infection, UTI = urinary tract infection, VIRAL = viral infection; **Healthcare exposures** (green bubbles): DIAL = outpatient dialysis, ED visit = emergency department visit without hospitalization, ELECT HOSP = non-emergent hospitalization, EMERG HOSP = emergent hospitalization, 2+ HOSP YEAR = at least two hospitalizations in the last year; INV = any invasive procedure, OUTPT INV, outpatient invasive procedure, SURG = surgery; Yes NH / No SNF = nursing home residents without a skilled nursing facility exposure within 3 months of CDI; No NH / Yes SNF = people without a nursing home exposure but with a skilled nursing facility exposure within 3 months of CDI **Age** (clear bubbles): 66–70 = 66 to 70 years old, 71–75 = 71 to 75 years old, 76–80 = 76 to 80 years old, 81–85 = 81 to 85 years old, 85+ = >85 years old)

There were 1,465,927 persons in the Medicare data with a total of 488,344 hospitalizations and 11,385,402 outpatient visits in 2009 (Table 1). The overall incidence of CDI per 100,000 persons was 677. The prevalence of comorbidities, and CDI incidence and PAR% for each comorbidity are in Table A in S1 File and Figs 1 and 2. None of the comorbidities in the top three of CDI incidence (weight loss 4,124/100,000, paralysis 4,011/100,000, blood loss anemia 3,305/100,000) were in the top three for PAR% because of the relatively low prevalence of these conditions. The comorbidities in the top three for PAR% were hypertension (60.6%), deficiency anemia (37.9%), and congestive heart failure (30.2%). 793,466 (54.1%) of people had hypertension. Discarding hypertension, fluid and electrolyte disorder would be in the top three PAR% (29.6%), and the prevalence of these three comorbidities with a top PAR% would be lower than 13%.

**Table 1:** Characteristics of the study population.

Characteristic	Number (%)
Population size	1,465,927
Female	897,227 (61.2)
White race	1,266,929 (86.4)
Number of people with ≥ 1 hospitalization	279,797 (19.1%)
Total number of hospitalizations	488,344
Number of people with ≥ 1 outpatient encounter	1,297,518 (88.5%)
Number of outpatient visits	11,385,402
Number of people with 1 or more episodes of CDI	9,401 (0.64%)
Incidence of CDI/100,000 persons (≥ 1 episode)*	677

* Person-year exposure was censored for death

The incidence of CDI and PAR% were also calculated for a variety of infections and healthcare exposures. As more time elapsed between the infections and healthcare exposures examined and the index date, in general the CDI incidence and PAR% decreased, indicating most CDI cases occurred within three months of the inciting infection or healthcare exposure (Table A in S1 File). As a result, the focus of the infection and healthcare exposures were on the results within three months before the index date. The relative changes over time in CDI incidence and PAR% were lowest for “chronic” health exposures, i.e. outpatient dialysis and nursing home residence.

The incidence of CDI was highest for several acute infections (Table A in S1 File and Figs 1 and 2). Less than 25% of Medicare beneficiaries had one or more infections within three months before the index date, but the PAR% for people with at least one infection was 74·4%. Only one infection in the top three for CDI incidence (septicemia 19,710/100,000, clinical sepsis 16,825/100,000, and gastroenteritis 13,239/100,000) was in the top three for PAR% (septicemia, 24.8%). The other two infections in the top three for PAR% were urinary tract infection (40.5%) and pneumonia (35.2%). The prevalence of all top three PAR% infections was lower than 8.0%.

More frequent healthcare exposures and healthcare exposures associated with greater acuity of illness were associated with a higher CDI incidence. The CDI incidence was higher if the person had at least two hospitalizations in the past year (4,555/100,000 persons) compared to one hospitalization (1,561/100,000 persons) (Table A in S1 File, Figs 1 and 2). The CDI incidence was also higher for emergent hospitalizations within the past three months (6,594/100,000 persons) than for non-emergent hospitalizations (2,989/100,000 persons) and treat-and-release ED visits (2,840/100,000 persons). The healthcare exposure in the past three months with the highest CDI incidence was among people living in the community who were admitted to a skilled nursing facility after a hospitalization (no NH / yes SNF 7,762/100,000), followed by emergent hospitalization, and surgery (6,172/100,000). The healthcare exposures in the top three for PAR% were any hospitalization in the last year (70.5%), an emergent hospitalization in the past three months (48.5%), and invasive procedure in the past three months (38.9%).

**Fig 3:**
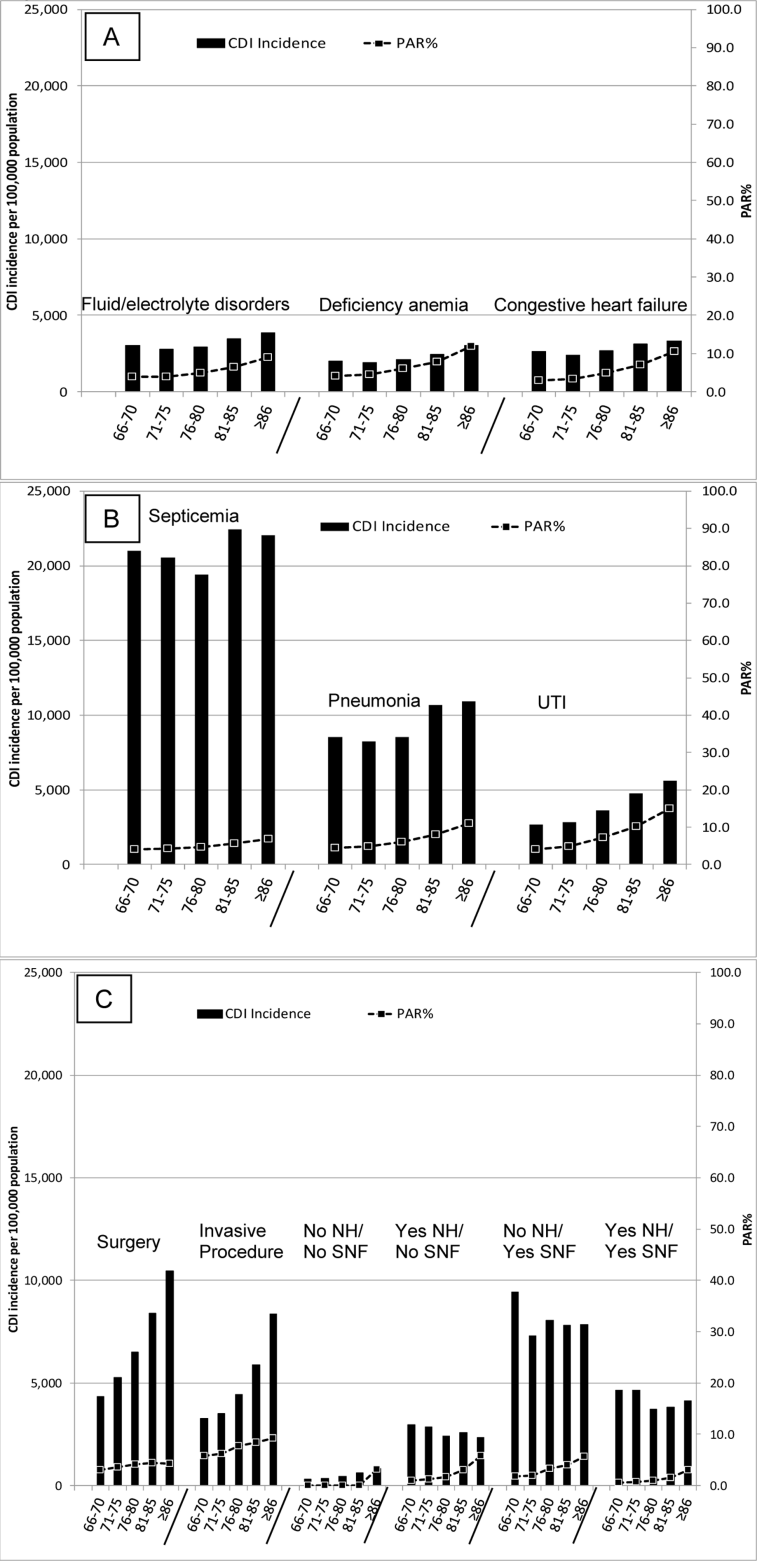
CDI incidence and CDI population attributable risk percent (PAR%) of comorbidities (A), infections within 3 months of CDI (B), and healthcare exposures within 3 months of CDI (C) stratified by age. Comorbidities and infections were limited to those with a top three PAR%. No NH / No SNF = people without a nursing home or skilled nursing facility exposure within 3 months of CDI; Yes NH / No SNF = nursing home residents without a skilled nursing facility exposure within 3 months of CDI; No NH / Yes SNF = people without a nursing home exposure but with a skilled nursing facility exposure within 3 months of CDI; Yes NH / Yes SNF = nursing home residents.

**Fig 4:**
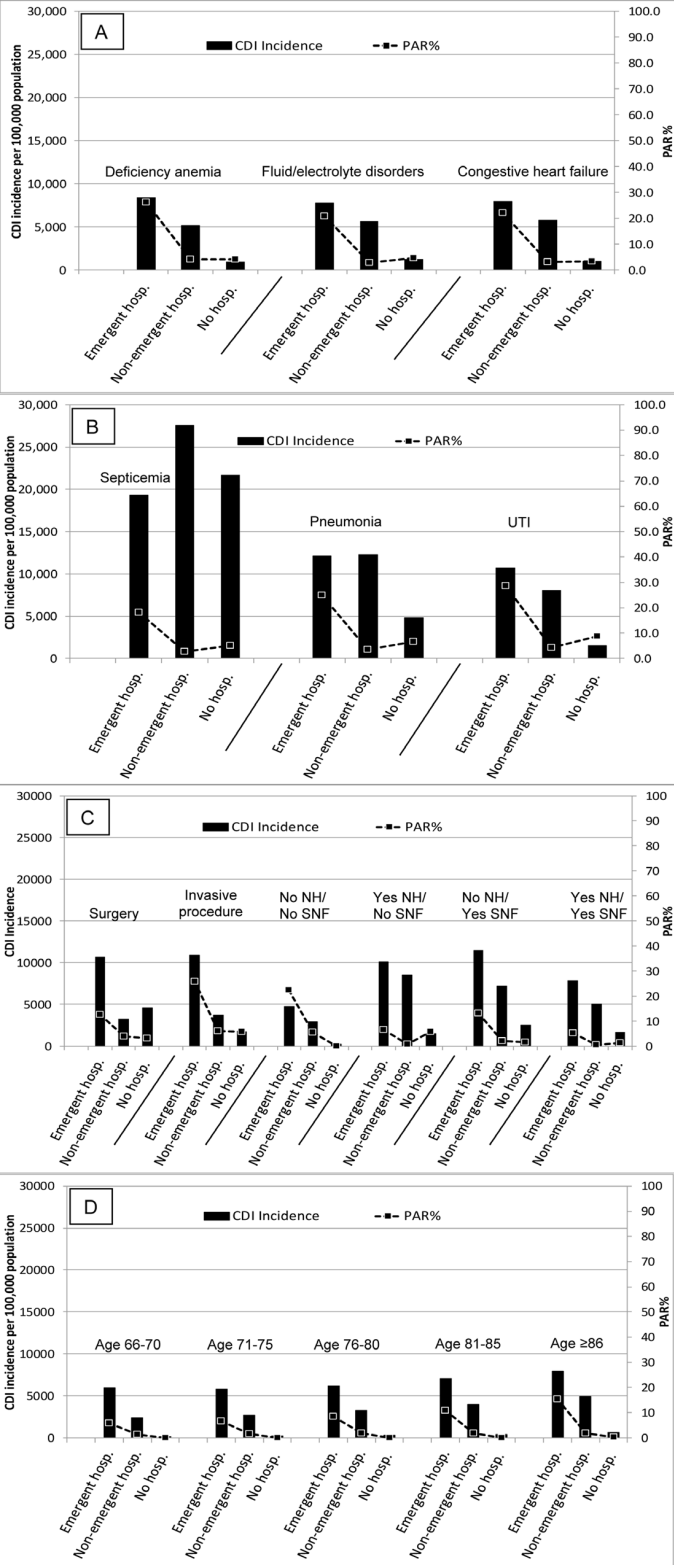
CDI incidence and CDI population attributable risk percent (PAR%) of comorbidities (A), infections within 3 months of CDI (B), healthcare exposures within 3 months of CDI (C), and age stratified by hospital exposures (D) in the three months prior to CDI. Comorbidities and infections were limited to those with a top three PAR%. No NH / No SNF = people without a nursing home or skilled nursing facility exposure within 3 months of CDI; Yes NH / No SNF = nursing home residents without a skilled nursing facility exposure within 3 months of CDI; No NH / Yes SNF = people without a nursing home exposure but with a skilled nursing facility exposure within 3 months of CDI; Yes NH / Yes SNF = nursing home residents

The CDI incidence by age was lower than the overall CDI incidence for all age groups other than 81–85 and >85 years old (Fig 2, Table A in SI File). Only the >85 age group had a CDI incidence greater than any individual comorbidity irrespective of the age. Likewise, the only infection with a lower CDI incidence than these two age groups was otitis media, and all healthcare exposures had a higher CDI incidence than all age groups. Stratifying all comorbidities, infections, and healthcare exposures by age and hospital exposures in the past three months indicate hospital exposure is a better marker to identify populations at risk for CDI than age, with emergent hospitalization being the key exposures (Figs 3 and 4, Tables B and C in S1 File). In general, CDI incidence and PAR% increase with increasing age, but the proportional differences in CDI incidence and PAR% for emergent hospitalization versus non-emergent hospitalization and no hospitalization is much greater than between the age categories.

## Discussion

The purpose of this study was to develop a more complete understanding of specific subpopulations at risk for CDI. These are essential data needed to design targeted CDI prevention strategies and to develop and assess CDI control policies that may impact CDI risk in the population. We used an innovative approach to identify target populations for CDI prevention interventions, focusing not just on the incidence of CDI within certain subpopulations, but also on the PAR%, which takes into account both the incidence of disease (i.e. CDI) and the prevalence of exposures/subpopulations (i.e. comorbidities, acute infections, healthcare utilization) within the population. PAR% was instrumental in determining the impact of smoking on lung cancer risk, leading to targeting of prevention efforts to reduce lung cancer by reducing smoking [18,19]. From the perspective of identifying populations to target for surveillance and prevention efforts, focus on a combination of high incidence and high PAR% exposures is likely to be most effective, since surveillance and prevention efforts will be focused not just on populations most likely to benefit (i.e. high incidence), but also on exposures that account for a high proportion of CDI cases [19].

Among the many informative findings of this study, the primary one is that markers of overall debility (i.e. deficiency anemia and fluid and electolyte disorders) and acuity of illness, in addition to recent infections, are keys to identifying populations at risk for CDI. The CDI incidence of the comorbidities was relatively modest compared to infections and healthcare exposures, and none of the comorbidities typically found to have strong associations with CDI in the hospital setting (e.g. congestive heart failure, cancer, renal failure, chronic lung disease) were amongst the conditions with the highest CDI incidence [20,21]. Conversely, deficiency anemia and fluid and electrolyte disorders, indicators of overall debility, were among the comorbidities with the highest PAR%.

The importance of overall debility and acuity of illness in identification of populations at higher risk for CDI is further supported by the healthcare exposure and age results. The healthcare exposures with the highest CDI incidence were those associated not only with a recent hospitalization, but a recent emergent hospitalization. The PAR% for an emergent hospitalization within the three months prior to CDI was almost 50%. For all comorbidities and healthcare exposures, the CDI incidence and PAR% were higher for people with a recent emergent hospitalization compared to a non-emergent hospitalization. This is likely a reflection that people with emergent hospitalizations have a high severity of underlying illness due to an acute condition and often have infections and/or are at increased risk for developing infections. In contrast, people with non-emergent hospitalizations are typically admitted for elective procedures, and therefore are healthy enough for the elective procedure. Consistent with this, this trend did not always hold when comparing non-emergent hospitalizations to people who had no hospitalization, again indicating it is important to take into account the circumstances surrounding the hospitalization when identifying people at high risk for CDI.

At face-value, it may seem counter intuitive that age by itself may not be an ideal marker to identify populations at risk for CDI among the elderly. A dose-response relationship between age and CDI incidence and PAR% exists, but this relationship was not as strong as the association between CDI and emergent hospitalization or CDI and infection. In this study there were 213,500 people >85 years old, the age category with the highest CDI incidence (1,330/100,000) and PAR% (18.3%). In comparison, there were 73,779 people with an emergent hospitalization (6,514/100,000 and 48.5% CDI incidence and PAR%, respectively) and 117,127 people with a urinary tract infection (3,632/100,000 and 40.5%, respectively). Targeting CDI prevent efforts to these two groups would prevent more CDI than targeting people greater than 85 years old, but fewer people would be need to be exposed to the intervention.

The conditions associated with the highest CDI incidence not surprisingly were acute infections. Six of the infections evaluated had a CDI incidence higher than the healthcare exposure with the highest CDI incidence (Fig 1, Table A in S1 File). Infections are associated with a high acuity of illness, and people who are debilitated are at increased risk for infection. In addition, antimicrobials are used to treat infections, and antimicrobial exposure is indisputably the greatest risk factor for CDI [22]. The association of CDI with previous gastroenteritis is less clear. We could not determine from the administrative data alone if the previous episode coded as gastroenteritis was actually undiagnosed CDI, or if gastroenteritis was due to another cause and asymptomatic *C*. *difficile* colonization was subsequently detected and coded as CDI [23]. Alternatively, some data indicate gastroenteritis due to causes other than *C*. *difficile* may predispose to CDI [24]. Two infections of particular note from our study are pneumonia and urinary tract infection. These infections had the highest PAR% (35.2% and 40.5%, respectively) among acute infections, and are commonly treated with cephalosporins and fluoroquinolones, antimicrobials associated with the highest risk for CDI [22].

This study utilized the Medicare 5% random sample, an administrative claims database. Advantages are that the data are collected in a similar fashion and the cohorts are large and representative of the US population >65 years of age, and use of administrative data to identify CDI has been validated [25]. Limitations include reliance on coding data without an assessment of the patient’s clinical course. Efforts were made to minimize risk of temporally misclassifying an event that occurred after CDI as an exposure. In addition, the Medicare 5% random sample lacks data on inpatient antimicrobial exposures, outpatient antimicrobial exposures among people who do not have part D, and laboratory results and vital signs in all settings. Availability of antimicrobial exposure and vital sign data would help identify the impact of specific antimicrobials and acuity of illness on CDI incidence and PAR%. The results of this study may not be generalizable outside of the US as well.

A major strength of analysis of the Medicare 5% random sample is that these data allowed for a population-based approach to identify specific subpopulations at high risk for CDI. This is particularly important as <25% of CDI cases have onset within a hospital [4]. Also, identifying individuals at risk for CDI based on a multitude of risk factors is complex, not always feasible operationally, and it is not always clear what to do once the individual is identified [26]. A population based approach allows for focus on easily identifiable characteristics, which can facilitate implementation of subpopulation-based CDI prevention interventions.

A unique feature of this study was use of PAR% in addition to CDI incidence to identify subpopulations at risk for CDI. When used in combination, these epidemiological tools can be leveraged to identify key subpopulations to target interventions. This is demonstrated visually with Fig 2, where potential desirable target populations can be quickly identified based on the size of the population (x-axis), incidence of CDI (y-axis), and size of the bubble (PAR%). In some respects we applied PAR% outside of its traditional context, in which there is a direct cause and effect relationship between the exposure and outcome being examined (e.g. smoking and lung cancer). The PAR% represents the anticipated decrease in the outcome if the exposure were completely removed from the population. Along these lines, the subpopulations were analyzed without risk adjustment of the PAR% for a subpopulation. The goal of this study was to determine if there were readily identifiable subpopulations to which prevention efforts could be targeted. Risk adjustment is not necessary to accomplish this, and would make identification of subpopulations significantly more complicated.

The broader context we applied to the calculation of PAR% for CDI was done purposefully. There is a *C*. *difficile* vaccine currently in phase III study, with others in development [27]. Although deficiency anemia is unlikely to be in the causal pathway for CDI, it is likely a marker for people at increased risk for an emergent hospitalization and/or antimicrobial exposure, and therefore may be a marker to identify populations where there are both individual (high incidence condition) as well as population (high PAR%) benefits to vaccination. Examples of other high-value targets for vaccination we identified would be emergent hospitalizations, pneumonia, and urinary tract infections. The results of this study can also be used to design and implement other CDI prevention interventions. Antimicrobials are frequently initiated in the ED, and the PAR% for treat and release ED visits was 22.3%. Antimicrobial stewardship programs typically focus on the hospital setting, but including the ED in antimicrobial stewardship activities may be beneficial in regards to preventing CDI. The same argument applies to antimicrobial stewardship programs that target pneumonia and urinary tract infection diagnosis and treatment, which could lead to large reductions in CDI incidence in the population because of the high PAR% for these infections.

Past research has focused on CDI incidence and independent risk factors for CDI. Although this approach will identify subpopulations at increased CDI risk, it may miss opportunities to maximize CDI prevention efforts by not taking into account the prevalence of the risk factors. The PAR% adds to our understanding by identifying specific subpopulations that account for a large proportion of CDI cases. Attributable risk measures provide a public health dimension to the appraisal of risks and assist in maximizing the effectiveness of prevention efforts by focusing on not just high risk but highly exposed populations [19,28]. Using the combination of exposure prevalence, CDI incidence, and PAR%, we found that many populations often considered targets for CDI prevention account for a relatively low proportion of total CDI cases. Based on the Medicare data, specific subpopulations that are easily identifiable could be targeted for high-impact, cost-effective CDI prevention interventions. These potential high impact subpopulations include people with deficiency anemia, congestive heart failure, fluid and electrolyte disorders, urinary tract infections, pneumonia, emergency department visits (in particular if it leads to a hospitalization), and/or invasive procedures. Necessary next steps are to design and implement interventions to prevent CDI based upon targeting these populations.

## Supporting Information

S1 File Database descriptions. Table A in SI File: Prevalence of comorbidities, infections, healthcare exposures, and age groups; CDI incidence and population attributable risk percent (PAR%) of comorbidities, infections, healthcare exposures, and age groups. Table B in S1 File: Prevalence of comorbidities, infections within 3 months of CDI, and healthcare exposures within 3 months of CDI and CDI incidence and population attributable risk percent (PAR%) of comorbidities, infections within 3 months of CDI, and healthcare exposures within 3 months of CDI stratified by age. Table C in S1 File: Prevalence of comorbidities, infections within 3 months of CDI, and healthcare exposures within 3 months of CDI and CDI incidence and population attributable risk percent (PAR%) of comorbidities, infections, healthcare exposures, and age stratified by hospital exposure within 3 months of CDI.
(DOCX)
