# Supplementary material for: Identification of Medicare Recipients at Highest Risk for Clostridium difficile Infection in the US by Population Attributable Risk Analysis
Source: PLoS One. 2016 Feb 9;11(2):e0146822. doi: 10.1371/journal.pone.0146822 (PMC4747338; doi:10.1371/journal.pone.0146822)
Supplement: S1 File — Table A in SI File: Prevalence of comorbidities, infections, healthcare exposures, and age groups; CDI incidence and population attributable risk percent (PAR%) of comorbidities, infections, healthcare exposures, and age groups. Table B in S1 File: Prevalence of comorbidities, infections within 3 months of CDI, and healthcare exposures within 3 months of CDI and CDI incidence and population attributable risk percent (PAR%) of comorbidities, infections within 3 months of CDI, and healthcare exposures within 3 months of CDI stratified by age. Table C in S1 File: Prevalence of comorbidities, infections within 3 months of CDI, and healthcare exposures within 3 months of CDI and CDI incidence and population attributable risk percent (PAR%) of comorbidities, infections, healthcare exposures, and age stratified by hospital exposure within 3 months of CDI. (DOCX) [file pone.0146822.s001.docx]

**Supplementary materials.**

**Database descriptions**

*Medicare 5% random sample:* The Medicare 5% random sample files used included Inpatient, Carrier Claims (physicians and other medical services), Outpatient (outpatient facility services), Beneficiary Summary, Skilled Nursing Facility, and Part D Drug Event. The files contain claims data with all ICD-9-CM diagnosis and procedure codes, Healthcare Common Procedure Coding System (HCPCS) codes, dates of service, and beneficiary Medicare enrollment and demographic data. The Part D drug event file contains the National Drug Code (NDC) to identify outpatient drug utilization, including quantity dispensed, days supplied, route/dosage, and financial information. Medicare Part A and Part B fee-for-service coverage from January 1, 2008 through December 31, 2009 (or death) was required.

**Date of Onset of CDI**

The date of onset of CDI was defined as the first date with a coded diagnosis of CDI, unless additional information was available to define an earlier date as the date of onset. The additional information used to define an earlier date of symptom onset was a diagnosis of diarrhea (ICD-9-CM diagnosis codes 009.0-009.3, 787.91), abdominal pain (789.00-789.09) or nausea (787.01, 787.02), or CPT-4 codes in outpatient files for stool microbiology tests (87045, 87046, 87177, 87269, 87329, 87272, 87328, 87335, 87427, 87425). If any of these were present within the 28 days before the CDI diagnosis code, the date of onset was the earliest date of the CDI indicator.

For persons with CDI in the first quarter of 2009, 2008 billing/claims data were queried for evidence of CDI in the previous 84 days; if present the first episode in 2009 was excluded to avoid misclassifying a recurrent CDI episode or carry-forward of the CDI ICD-9-CM diagnosis code as an initial episode of CDI. These persons were eligible to have an initial episode of CDI later in 2009 if there was a subsequent 84-day period during which the person did not meet criteria for CDI.

**Comorbidities, Prior Infections, and Healthcare Exposures:**

The year prior to the index date was used to identify comorbidities, acute infections, and healthcare exposures. Chronic comorbidities were defined using the algorithm of Elixhauser, with modification for claims data according to Klabunde et al (1;2). We included index hospitalizations in the identification of all comorbidities except fluid and electrolyte disorders and weight loss, since those conditions may have been due to CDI.

For acute infections diagnosed in an outpatient setting, only a single claim was required, excluding laboratory claims. Acute infections during the index CDI hospitalization were also included if the acute infection was the primary inpatient diagnosis and the CDI was determined to be hospital-onset (see supplementary materials). Date of previously coded hospital exposures and infections were based on the date of hospital discharge.

Surgical procedures prior to the index date were identified from ICD-9-CM procedure codes and Current Procedural Terminology (CPT) codes using a modification of the National Healthcare Safety Network (NHSN) procedure code list (see supplementary materials). Outpatient and inpatient invasive procedures were identified using UB-92 revenue codes for operating room expenses. Outpatient procedures were identified to match those of Chitnis et al as closely as possible (3). Outpatient dialysis was defined by UB-92 revenue codes, ICD-9-CM diagnosis and CPT codes, and outpatient colonoscopy was defined by CPT codes (see supplementary materials).

Prior hospitalizations were categorized as emergent if the hospitalization originated in the emergency department (ED, i.e., UB-92 revenue codes 0450-0459). Treat-and-release ED visits were defined by UB-92 revenue codes 0450-0459 in the outpatient claims files. Nursing home residents were identified by the method of Yun et al (4). Once a person met criteria as a nursing home resident, they were considered a nursing home resident from that point forward. Nursing home and skilled nursing facility exposures were split into four categories based on the exposures within 3 months of the index date: not a nursing home resident and no skilled nursing exposures (no NH/no SNF); not a nursing home resident with a skilled nursing exposure (no NH / yes SNF); nursing home resident with a skilled nursing exposure (yes NH / yes SNF); and nursing home resident with no skilled nursing exposures (yes NH / no SNF). These groups were created as all patients with a recent SNF had a hospitalization within 100 days of admission to the SNF, and risk for CDI is greater among patients in a SNF setting than residential nursing home setting (5).

I. ICD-9-CM codes used to define comorbidities

|  | | **ICD-9-CM diagnosis code** |
| --- | --- | --- |
| **Comorbidities** |  | |
| Alcohol abuse | 291.0–291.3, 291.5–291.9, 303.00–303.93, 305.00–305.03 | |
|  |  | |
| Blood loss anemia | 280.0 | |
| Cancer– lymphoma | 200.00–202.38, 202.50–203.82, 238.6, 273.3 | |
| Cancer– metastatic cancer | 196.0–199.1, 209.70–209.79, 789.51 | |
| Cancer– solid tumor | 140.0–172.9, 174.0–175.9, 179-195.8, 209.00–209.36, 258.01–258.03 | |
| Chronic Pulmonary disease | 490-505, 506.4 | |
| Coagulopathy | 286.0–286.9, 287.1, 287.3–287.5, 289.84 | |
| Congestive heart failure | 398.91, 402.01, 402.11, 402.91, 404.01, 404.03, 404.11, 404.13, 404.91, 404.93, 428.0–428.9 | |
| Deficiency anemias | 280.1–281.9, 285.21–285.29, 285.9 | |
| Depression | 300.4, 301.12, 309.00, 309.1, 311 | |
|  |  | |
| Diabetes mellitus | 249.00–249.91, 250.00–250.93 | |
| Fluid/electrolyte disorders | 276.0-276.9 | |
| Hypertension | 401.0, 401.1, 401.9, 402.00–405.99, 437.2 | |
| Hypothyroidism | 243-244.2, 244.8, 244.9 | |
| Liver diseases | 070.22, 070.23, 070.32, 070.33, 070.44, 070.54, 456.0–456.21, 571.0, 571.2–571.9, 572.3, 572.8, 573.5, V42.7 | |
| Malnutrition/ weight loss | 260–263.9, 783.21, 783.22 | |
| Neurologic disorders | 330.1- 332.0, 333.4, 333.5, 333.71-333.79, 333.85, 333.94, 334.0-335.9, 338.0, 340, 341.1-341.9, 345.00- 345.91, 347.00-347.11, 768.70-768.72, 780.31-780.39, 780.97, 784.3 | |
| Obesity | 278.00, 278.01, 278.03, 793.91, V85.30–V85.45 | |
| Paralysis | 342.0-344.9, 438.20-438.53, 780.72 | |
| Peripheral vascular disease | 440.0–440.9, 441.00–441.9, 442.0–442.9, 443.1–443.9, 444.21, 444.22, 447.1, 449, 557.1, 557.9, V43.4 | |
| Pulmonary circulatory disorders | 415.11-416.9, 417.9 | |
| Psychoses | 295.00–298.9, 299.10, 299.11 | |
| Renal failure | 403.01, 403.11, 403.91, 404.02, 404.03, 404.12, 404.13, 404.92, 404.93, 585.3–586, V42.0, V45.1–V45.12, V56.0–V56.8 | |
| Rheumatoid arthritis/collagen vascular diseases | 710.0-710.9, 714.0-714.9, 720.0-720.9, 725 | |
| Valvular disease | 093.20-093.24, 394.0-397.9, 424.0-424.99, 746.3-746.6, V42.2, V43.3 | |

1. **ICD-9-CM codes used to define infection variables**

| **Infection** | **ICD-9-CM diagnosis codes** |
| --- | --- |
| Septicemia | 003.1, 020.2, 022.3, 036.2, 036.42, 038.0, 038.1, 038.10, 038.11, 038.12, 038.19, 038.2, 038.3, 038.4, 038.40, 038.41, 038.42, 038.43, 038.44, 038.49, 038.8, 038.9, 040.82, 449, 421.0, 421.1, 421.9, 422.92, 790.7 |
| Clinical sepsis | 995.91, 995.92, 785.52 |
| Pneumonia | 003.22, 020.3, 020.4, 020.5, 021.2, 022.1, 039.1, 073.0, 073.7, 073.8, 073.9, 083.0, 480.0, 480.1, 480.2, 480.3, 480.8, 480.9, 481, 482.0, 482.1, 482.2, 482.3, 482.30, 482.31, 482.32, 482.39, 482.4, 482.40, 482.41, 482.42, 482.49, 482.8, 482.81, 482.82, 482.83, 482.84, 482.89, 482.9, 483, 483.0, 483.1, 483.8, 484, 484.1, 484.3, 484.5, 484.6, 484.7, 484.8,  485, 486, 510.0, 510.9, 513.0, 997.31 |
| Urinary tract infection (UTI) | 032.84, 590.00, 590.01, 590.1, 590.10, 590.11, 590.2, 590.3, 590.80, 590.81, 590.9, 595.0, 595.2, 595.89, 595.9, 597.0, 598.00, 598.01, 599.0, 601.0, 601.1, 601.2, 601.3, 601.4, 601.9, 603.1, 604.0, 607.2, 608.0, 608.4, 646.60, 646.61, 646.62, 646.63, 646.64, 996.64 |
| Skin and soft tissue infection (SSTI) | 020.1, 021.0, 022.0, 032.85, 035, 039.0, 039.3, 039.4, 039.8, 039.9, 040.0, 040.1, 040.2, 040.3, 040.42, 040.81, 078.3, 082.0, 082.1, 082.2, 082.3, 082.40, 082.41, 082.49, 082.8, 082.9, 083.0, 083.1, 083.2, 083.8, 083.9, 087.0, 087.1, 087.9, 088.0, 088.81, 088.82, 088.89, 088.9, 098.50, 567.31, 680.0, 680.1, 680.2, 680.3, 680.4, 680.5, 680.6, 680.7, 680.8, 680.9, 681.00, 681.01, 681.02, 681.10, 681.11, 681.9, 682.0, 682.1, 682.2, 682.3, 682.4, 682.5, 682.6, 682.7, 682.8, 682.9, 683, 684, 685.0, 686.0, 686.00, 686.01, 686.09, 686.1, 686.8, 686.9, 675.00, 675.01, 675.02, 675.03, 675.04, 675.10, 675.11, 675.12, 675.13, 675.14, 675.80, 675.81, 675.82, 675.83, 675.84, 675.90, 675.91, 675.92, 675.93, 675.94, 705.83, 727.89, 7280, 728.86 |
| Surgical site infection (SSI) | 9985, 998.51, 998.59, 996.60, 996.61, 996.62, 996.63, 996.65, 996.66, 996.67, 996.68, 996.69 |
| Bone infection | 003.23, 003.24, 0261, 036.82, 098.50, 098.51, 098.52, 098.53, 098.59, 376.03, 513.1, 519.2, 711.00, 711.01, 711.02, 711.03, 711.04, 711.05, 711.06, 711.07, 711.08, 711.09, 711.90, 711.91, 711.91, 711.92, 711.93, 711.94, 711.95, 711.96, 711.97, 711.98, 711.99, 730.00, 730.01, 730.02, 730.03, 730.04, 730.05, 730.06, 730.07, 730.08, 730.09, 730.10, 730.11, 730.12, 730.13, 730.14, 730.15, 730.16, 730.17, 730.18, 730.19, 730.20, 730.21, 730.22, 730.23, 730.24, 730.25, 730.26, 730.27, 730.28, 730.29, 730.30, 730.31, 730.32, 730.33, 730.34, 730.35, 730.36, 730.37, 730.38, 730.39, 730.80, 730.81, 730.82, 730.83, 730.84, 730.85, 730.86, 730.87, 730.88, 730.89, 730.90, 730.91, 730.92, 730.93, 730.94, 730.95, 730.96, 730.97, 730.98, 730.99 |
| Organ infection | 003.21, 032.82, 036.0, 036.1, 036.3, 036.40, 036.41, 036.43, 036.89, 091.81, 100.81, 245.0, 253.8, 254.1, 289.59, 320.0, 320.1, 320.2, 320.3, 320.7, 320.8, 320.81, 320.82, 320.89, 320.9, 322.9, 323.1, 323.4, 323.41, 323.42, 324.0, 324.1, 324.9, 420, 420.0, 420.90, 420.99 |
| Female pelvic infection | 091.0, 091.1, 091.2, 091.3, 091.4, 091.50, 091.51, 091.52, 091.61, 091.62, 091.69, 091.7, 091.82, 091.89, 091.9, 092.0, 092.9, 098.0, 098.10, 098.11, 098.12, 098.13, 098.14, 098.15, 098.16, 098.17, 098.19, 0982, 098.30, 098.31, 098.32, 098.33, 098.34, 098.35, 098.36, 098.37, 098.39, 098.40, 098.41, 098.42, 098.43, 098.49, 098.7, 098.81, 098.82, 098.83, 098.84, 098.85, 098.86, 098.89, 099.0, 099.1, 099.2, 099.3, 099.4, 099.40, 099.41, 099.49, 099.50, 099.51, 099.52, 099.53, 099.54, 099.55, 099.56, 099.59, 099.8, 099.9, 597.80, 597.81, 597.89, 614.0, 614.1, 614.2, 614.3, 614.4, 614.5, 614.7, 614.9, 615.0, 615.1, 615.9, 616.0, 616.10, 616.3, 616.4, 634.00, 634.01, 634.02, 635.00, 635.01, 635.02, 636.00, 636.01, 636.02, 637.00, 637.01, 637.02, 638.00, 638.01, 638.02, 639.0, 646.60, 646.61, 646.62, 646.63, 646.64, 647.00, 647.01, 647.02, 647.03, 647.04, 647.10, 647.11, 647.12, 647.13, 647.14, 647.20, 647.21, 647.22, 647.23, 647.24, 647.80, 647.81, 647.82, 647.83, 647.84, 658.40, 658.41, 658.43, 659.30, 659.31, 659.33, 670.00, 670.02, 670.04, 670.10, 670.12, 670.14, 670.20, 670.22, 670.24, 670.30, 670.32, 670.34, 670.80, 670.82, 670.84 |
| Gastroenteritis | 001.0, 001.1, 001.9, 002.0, 002.1, 002.2, 002.3, 002.9, 003.0, 003.20, 003.29, 003.8, 003.9, 004.0, 004.1, 004.2, 004.3, 004.8, 004.9, 005.0, 005.1, 005.2, 005.3, 005.4, 005.8, 005.81, 005.89, 005.9, 008.0, 008.00, 008.01, 008.02, 008.03, 008.04, 008.09, 008.1, 008.2, 008.3, 008.41, 008.42, 008.43, 008.44, 008.46, 008.47, 008.49, 008.5, 009.0, 009.1, 009.2, 009.3, 021.1, 022.2 |
| Intraabdominal abscess / peritonitis | 032.83, 039.2, 530.19, 530.86, 536.41, 540.0, 540.1, 540.9, 562.00, 562.01, 562.02, 562.03, 562.10, 562.11, 562.12, 562.13, 566, 567.0, 567.1, 567.2, 567.21, 567.22, 567.23, 567.29, 567.38, 567.39, 567.9, 569.5, 569.61, 572.0, 574.00, 574.01, 574.30, 574.31, 574.60, 574.61, 574.80, 574.81, 575.0, 575.12, 577.0 |
| Upper respiratory tract infection | 460, 461.0, 461.1, 461.2, 461.3, 461.8, 461.9, 462, 464.0, 464.00, 464.01, 464.10, 464.11, 464.20, 464.21, 464.30, 464.31, 464.4, 464.50, 464.51, 465.0, 465.8, 465.9, 466.0, 466.11, 466.19, 473.0, 473.1, 473.2, 473.3, 473.8, 473.9, 476.0, 476.1 |
| Tonsillitis | 032.0, 032.1, 032.2, 032.3, 032.81, 032.89, 032.9, 033.0, 033.1, 033.8, 033.9, 034.0,  034.1, 076.0, 076.1, 076.9, 098.6, 370.55, 376.01, 376.02, 376.03, 383.20, 383.21, 383.22, 463, 474.0, 474.00, 474.01, 474.02, 475, 478.19, 478.21, 478.22, 478.24, 478.29, 4785, 478.71, 478.79, 4789, 519.01 |
| Otitis | 380.10, 380.11, 380.13, 380.14, 380.16, 381.00, 381.01, 381.02, 381.03, 381.10, 381.19,  381.20, 381.29, 381.3, 381.4, 381.50, 381.51, 381.52, 382.0, 382.00, 382.01, 382.02, 382.3, 382.4, 382.9, 383.00, 383.01, 383.02, 383.1, 384.00, 384.01, 384.09, 384.1 |
| Oral infections | 522.0, 522.5, 522.6, 522.7, 523.00, 523.01, 523.10, 523.11, 523.30, 523.31, 523.32, 523.33, 523.40, 523.41, 523.42, 526.4, 527.3, 528.3, 528.5, 529.0 |
| Viral infections | 487, 488, 008.6, 008.8, 047, 048, 049.0, 049.1, 053.0, 054.72, 321.2, 049.8, 049.9, 052.0, 054.3, 055.0, 056.01, 058.21, 058.29, 061, 062.0, 062.1, 062.2, 062.3, 062.4, 062.5, 066.40, 066.41, 066.42, 066.49, 066.8, 066.9, 053.1, 053.2, 053.7, 053.8, 053.9, 054.0, 054.1, 054.2, 054.4, 054.5, 054.6, 052.1, 052.2, 052.7, 052.8, 052.9, 054.71, 054.73, 054.74, 054.79, 054.8, 054.9, 057, 055.2, 055.7, 055.8, 055.9, 058.8, 056.00, 056.79, 056.8, 056.9, 058.10, 058.11, 074, 079.2, 077, 077.0, 077.1, 077.2, 077.3, 077.4, 077.5, 077.6, 077.7, 077.8, 077.99, 075, 078.5, 079.0, 480.0, 079.1, 079.6, 466.11 |

1. **Administrative codes used to define surgical variables**

| **Operative procedure** | **Surgical variable** | **ICD-9-CM procedure codes** | **CPT-4 codes** |
| --- | --- | --- | --- |
| Abdominal aortic aneurysm repair | AAA | 38.34, 38.44, 38.64 | 34800, 34802, 34803, 34804, 34805, 34825, 34826, 34830, 34831, 34832, 35081, 35082, 35091, 35092, 35102, 35103, 0078T, 0079T, 0080T, 0081T |
| Limb amputation | AMP | 84.00, 84.01, 84.02, 84.03, 84.04, 84.05, 84.06, 84.07, 84.08, 84.09, 84.10, 84.11, 84.12, 84.13, 84.14, 84.15, 84.16, 84.17, 84.18, 84.19, 84.91 | 24900, 24920, 25900, 25905, 25907, 25909, 25915, 25920, 25927, 26910, 26951, 26952, 27290, 27295, 27590, 27591, 27592, 27598, 27880, 27881, 27882, 27888, 28800, 28805, 28810, 28820, 28825 |
| Appendix surgery | APPY | 47.01, 47.09, 47.2, 47.91, 47.92, 47.99 | 44900, 44950, 44960, 44970 |
| Shunt for dialysis | AVSD | 39.27, 39.42 | 36818, 36819, 36820, 36821, 36825, 36826, 36827, 36828, 36829, 36830, 36831, 36832, 36833, 36834, 36835 |
| Bile duct, liver, or pancreatic surgery | BILI | 50.0, 50.12, 50.14, 50.21, 50.22, 50.23, 50.25, 50.26, 50.29, 50.3, 50.4, 50.61, 50.69, 51.31, 51.32, 51.33, 51.34, 51.35, 51.36, 51.37, 51.39, 51.41, 51.42, 51.43, 51.49, 51.51, 51.59, 51.61, 51.62, 51.63, 51.69, 51.71, 51.72, 51.79, 51.81, 51.82, 51.83, 51.89, 51.91, 51.92, 51.93, 51.94, 51.95, 51.99, 52.09, 52.12, 52.22, 52.3, 52.4, 52.51, 52.52, 52.53, 52.59, 52.6, 52.7, 52.92, 52.95, 52.96, 52.99 | 47010, 47015, 47100, 47120, 47122, 47125, 47130, 47300, 47350, 47360, 47361, 47362, 47370, 47371, 47379, 47380, 47381, 47400, 47420, 47425, 47460, 48000, 48001, 48020, 48100, 48105, 48120, 48140, 48145, 48146, 48148, 48150, 48152, 48153, 48154, 48155, 48160, 47560-47561 |
| Breast surgery | BRST | 85.12, 85.20, 85.21, 85.22, 85.23, 85.31, 85.32, 85.33, 85.34, 85.35, 85.36, 85.41, 85.42, 85.43, 85.44, 85.45, 85.46, 85.47, 85.48, 85.50, 85.53, 85.54, 85.55, 85.6, 85.70, 85.71, 85.72, 85.73, 85.74, 85.75, 85.76, 85.79, 85.93, 85.94, 85.95, 85.96 | 19101, 19112, 19120, 19125, 19126, 19300, 19301, 19302, 19303, 19304, 19305,  19306, 19307, 19316, 19318, 19324, 19325, 19328, 19330, 19340, 19342, 19350, 19355, 19357, 19361, 19364, 19366, 19367, 19368, 19369, 19370, 19371, 19380, 38500, 38525, 38740, 38745 |
| Cardiac surgery | CARD | 35.00, 35.01, 35.02, 35.03, 35.04, 35.06, 35.08, 35.10, 35.11, 35.12, 35.13, 35.14, 35.20, 35.21, 35.22, 35.23, 35.24, 35.25, 35.26, 35.27, 35.28, 35.31, 35.32, 35.33, 35.34, 35.35, 35.39, 35.42, 35.50, 35.51, 35.53, 35.54, 35.60, 35.61, 35.62, 35.63, 35.70, 35.71, 35.72, 35.73, 35.81, 35.82, 35.83, 35.84, 35.91, 35.92, 35.93, 35.94, 35.95, 35.98, 35.99, 37.10, 37.11, 37.12, 37.31, 37.32, 37.33, 37.35, 37.36, 37.37, 37.41, 37.49, 37.60 | 33496, 33400-33417, 33420-33430, 33460-33468, 33470-33478, 33641-33694 |
| Coronary artery bypass graft with both chest and donor site incisions | CBGB | 36.10, 36.11, 36.12, 36.13, 36.14, 36.19 | 33510-33516, 33517-33523 |
| Coronary artery bypass graft with chest incision only | CBGC | 36.15, 36.16, 36.17, 36.2 | 33533-33548 |
| Carotid endarterectomy | CEA | 38.12 | 35390, 35301-35390, 35501-35510 |
| Gall bladder surgery | CHOL | 51.03, 51.04, 51.13, 51.21, 51.22, 51.23, 51.24 | 47480, 47562, 47563, 47564, 47570, 47600, 47605, 47610, 47612, 47620, 47720, 47740,  47420-47490 |
| Colon surgery | COLO | 17.31, 17.32, 17.33, 17.34, 17.35, 17.36, 17.39, 45.03, 45.26, 45.41,45.49, 45.50, 45.52, 45.7, 45.71, 45.72, 45.73, 45.74, 45.75, 45.76, 45.79, 45.8, 45.81, 45.82, 45.83, 45.90, 45.92, 45.93, 45.94, 45.95, 46.03, 46.04, 46.10, 46.11, 46.13, 46.14, 46.40, 46.42, 46.43, 46.50,46.52, 46.60, 46.63, 46.64, 46.75, 46.76, 46.91, 46.92, 46.94, 47.48, 47.70, 47.82 | 44025,44140, 44141, 44143, 44144, 44145, 44146, 44147, 44150, 44151, 44160, 44204, 44205, 44206, 44207,44208, 44210 |
| Craniotomy | CRAN | 01.12, 01.14, 01.20, 01.21, 01.22, 01.23, 01.24, 01.25, 01.26, 01.27, 01.28, 01.29, 01.31, 01.32, 01.39, 01.41, 01.42, 01.51, 01.52, 01.53, 01.59, 02.11, 02.12, 02.13, 02.14, 02.91, 02.92, 02.93, 07.51, 07.52, 07.53, 07.54, 07.59, 07.61, 07.62, 07.63, 07.64, 07.65, 07.68, 07.69, 07.71, 07.72, 07.79, 38.01, 38.11, 38.31, 38.41, 38.51, 38.61, 38.81, 39.28 | 61304-61576, 61580-61619, 61680-61711 |
| Cesarean section | CSEC | 74.0, 74.1, 74.2, 74.4, 74.91, 74.99 | 59525, 59510-59515 |
| Spinal fusion | FUSN | 81.00, 81.01, 81.02, 81.03, 81.04, 81.05, 81.06, 81.07, 81.08 | 22532-22819, 22840-22865 |
| Open reduction of fracture | FX | 79.21, 79.22, 79.25, 79.26, 79.31, 79.32, 79.35, 79.36, 79.51, 79.52, 79.55, 79.56 | 23615, 23616, 23630, 23660, 23670, 23680, 24515, 24516, 24538, 24545, 24546, 24575, 24579, 24582, 24586, 24587, 24615, 24635, 24665, 24666, 24685, 25337, 25515, 25525, 25526, 25545, 25574, 25575, 25606, 25607, 25608, 25609, 25628, 25645, 25652, 25670, 25676, 27236, 27244, 27245, 27248, 27253, 27254, 27258, 27259, 27269, 27283, 27506, 27507, 27511, 27513, 27514, 27519, 27524, 27535, 27536, 27540, 27756, 27758, 27759, 27766, 27769, 27784, 27792, 27814, 27822,  27823, 27826, 27827, 27828 |
| Gastric surgery | GAST | 43.0, 43.42, 43.49, 43.5, 43.6, 43.7, 43.81, 43.82, 43.89, 43.91, 43.99, 44.15, 44.21, 44.29, 44.31, 44.38, 44.39, 44.40, 44.41, 44.42, 44.49, 44.5, 44.61, 44.62, 44.63, 44.64, 44.65, 44.67, 44.68, 44.69, 44.91, 44.95, 44.96, 44.97, 44.98 | 43500, 43520, 43775, 43840, 43889,  43501-43510, 43605-43611, 43620-43634, 43644-43645, 43647-43648, 43770-43774, 43830-43832, 43842-43843, 43870-43880, 43881-43888, 43845-43865 |
| Herniorrhaphy | HER | 17.11, 17.12, 17.13, 17.21, 17.22, 17.23, 17.24, 53.00, 53.01, 53.02, 53.03, 53.04, 53.05, 53.10, 53.11, 53.12, 53.13, 53.14, 53.15, 53.16, 53.17, 53.21, 53.29, 53.31, 53.39, 53.41, 53.42, 53.43, 53.49, 53.51, 53.59, 53.61, 53.62, 53.63, 53.69 | 49491, 49492, 49495, 49496, 49500, 49501, 49505, 49507, 49520, 49521, 49525, 49540, 49550, 49553, 49555, 49557, 49560, 49561, 49565, 49566, 49568, 49570, 49572, 49580, 49582, 49585, 49587, 49590, 49650, 49651, 49652, 49653, 49654, 49655, 49656, 49657, 49659, 55540 |
| Hip prosthesis | HRPO | 00.70, 00.71, 00.72, 00.73, 00.85, 00.86, 00.87, 81.51, 81.52, 81.53 | 27090, 27091, 27120, 27122, 27125, 27130, 27132, 27134, 27137, 27138, 27299 |
| Heart transplant | HTP | 27.54, 37.51, 37.52, 37.53, 37.55 |  |
| Abdominal hysterectomy | HYST | 68.31, 68.39, 68.41, 68.49, 68.61, 68.69 | 58150, 58152, 58180, 58200, 58210, 58541, 58542, 58543, 58544, 58548, 58553, 58554, 58556, 58570, 58571, 58572, 58573, 58951 |
| Knee prosthesis | KPRO | 00.80, 00.81, 00.82, 00.83, 00.84, 81.54, 81.55 | 27438, 27440, 27441, 27442, 27443, 27445, 27446, 27447, 27486, 27487 |
| Kidney transplant | KTP | 55.61, 55.69 |  |
| Laminectomy | LAM | 03.01, 03.02, 03.09, 80.50, 80.51, 80.53, 80.54, 80.59, 84.60, 84.61, 84.62, 84.63, 84.64, 84.65, 84.66, 84.67, 84.68, 84.69, 84.80, 84.81, 84.82, 84.83, 84.84, 84.85 |  |
| Liver transplant | LTP | 50.51, 50.59 | 63001-63051, 63055-63066, 63170-63200, 63250-63295 |
| Neck surgery | NECK | 30.1, 30.21, 30.22, 30.29, 30.3, 30.4, 31.45, 40.40, 40.41, 40.42 | 31300, 31370, 31375, 31380, 31382, 31530, 31531, 31535, 31536, 31540, 31541, 31545, 31546, 31560, 31561, 31578, 31580, 31582, 31584, 31587, 31588, 31590,  31360-31368, 31390-31395 |
| Kidney surgery | NEPH | 55.01, 55.02, 55.11, 55.12, 55.24, 55.31, 55.32, 55.34, 55.35, 55.39, 55.4, 55.51, 55.52, 55.54, 55.91 | 50010, 50020, 50040, 50045, 50060, 50065, 50070, 50075, 50100, 50120, 50125, 50130, 50135, 50205, 50220, 50225, 50230, 50234, 50236, 50240, 50250, 50280, 50290,  50541-50545 |
| Ovarian surgery | OVRY | 65.01, 65.09, 65.12, 65.13, 65.21, 65.22, 65.23, 65.24, 65.25, 65.29, 65.31, 65.39, 65.41, 65.49, 65.51, 65.52, 65.53, 65.54, 65.61, 65.62, 65.63, 65.64, 65.71, 65.72, 65.73, 65.74, 65.75, 65.76, 65.79, 65.81, 65.89, 65.92, 65.93, 65.94, 65.95, 65.99 | 58660, 58662, 58720, 58800, 58805, 58920, 58940, 58950, 58952, 58957, 58958, 58990,  58661-58679, 58740-58770, 58820-58822, 58900-58940, 58943-58952 |
| Pacemaker surgery | PACE | 00.50, 00.51, 00.52, 00.53, 00.54, 17.51, 17.52, 37.70, 37.71, 37.72, 37.73, 37.74, 37.75, 37.76, 37.77, 37.79, 37.80, 37.81, 37.82, 37.83, 37.85, 37.86, 37.87, 37.89, 37.94, 37.95, 37.96, 37.97, 37.98, 37.99 | 33215, 33222, 33226, 33206-33208, 33210-33211, 33212-33213, 33216-33217, 33218-33220, 33224-33225, 33233-33237 |
| Prostate surgery | PRST | 60.12, 60.3, 60.4, 60.5, 60.61, 60.69 | 52601, 52630, 52640, 52647, 52648, 52649, 52700, 55801, 55810, 55812,  55815, 55821, 55831, 55840, 55842,  55845, 55866 |
| Peripheral vascular bypass surgery | PVBY | 39.29 | 35700, 35870, 34001-34203, 35480-35485, 35511-35525, 35526-35571, 35601-35616, 35621-35671, 35701-35860, 35875-35907 |
| Rectal surgery | REC | 48.25, 48.35, 48.40, 48.42, 48.43, 48.49, 48.50, 48.51, 48.52, 48.59, 48.61, 48.62, 48.63, 48.64, 48.65, 48.69, 48.74 | 45100, 45108, 45126, 45130, 45135, 45136, 45171, 45172, 45190, 45260, 46080, 46200, 46320, 45000-45020, 45110-45123,  46020-46060, 46250-46262, 46270-46288, 46700-46707, 46710-46712, 46715-46748, 46750-46947 |
| Refusion of spine | RFUSN | 81.30, 81.31, 81.32, 81.33, 81.34, 81.35, 81.36, 81.37, 81.38, 81.39 |  |
| Small bowel surgery | SB | 45.01, 45.02, 45.15, 45.31, 45.32, 45.33, 45.34, 45.51, 45.61, 45.62, 45.63, 45.91, 46.01, 46.02, 46.20, 46.21, 46.22, 46.23, 46.24, 46.31, 46.39, 46.41, 46.51, 46.71, 46.72, 46.73, 46.74, 46.93 | 44050, 44055, 44316, 44680, 44700, 44005-44021, 44110-44127, 44186-44187, 44202-44203, 44300-44314, 44602-44603, 44615-44661, 44800-44899 |
| Spleen surgery | SPLE | 41.2, 41.33, 41.41, 41.42, 41.43, 41.5, 41.93, 41.95, 41.99 | 38100, 38101, 38102, 38115,  38120-38129 |
| Thoracic surgery | THOR | 32.09, 32.1, 32.20, 32.21, 32.22, 32.23, 32.25, 32.26, 32.29, 32.30, 32.39, 32.41, 32.49, 32.50, 32.59, 32.6, 32.9, 33.0, 33.1, 33.20, 33.25, 33.28, 33.31, 33.32, 33.33, 33.34, 33.39, 33.41, 33.42, 33.43, 33.48, 33.49, 33.98, 33.99, 34.01, 34.02, 34.03, 34.06, 34.1, 34.20, 34.26, 34.3, 34.4, 34.51, 34.52, 34.59, 34.6, 34.81, 34.82, 34.83, 34.84, 34.89, 34.93, 34.99, 53.80, 53.81, 53.83, 53.84, 83.82 | 31613, 31614, 32035, 32036, 32095, 32402, 32540, 32665, 31600-31611, 31750-31786, 31780-31830, 32100-32225, 32310-32320, 32440-32504, 32601-32650, 32651-32657, 32658-32661, 32662-32664, 32800-32820, 32900-32906, 39520-39545 |
| Thyroid and/or parathyroid surgery | THYR | 06.02, 06.09, 06.12, 06.2, 06.31, 06.39, 06.4, 06.50, 06.51, 06.52, 06.6, 06.7, 06.81, 06.89, 06.91, 06.92, 06.93, 06.94, 06.95, 06.98, 06.99 | 60000, 60280, 60281, 60512,  60200-60240, 60252-60271, 60500-60505 |
| Vaginal hysterectomy | VHYS | 68.51, 68.59, 68.71, 68.79 | 58260-58294 |
| Ventricular shunt | VSHN | 02.21, 02.22, 02.31, 02.32, 02.33, 02.34, 02.35, 02.39, 02.42, 02.43, 54.95 | 62180-62223, 62225-62258 |
| Exploratory laparotomy | XLAP | 53.71, 53.72, 53.75, 54.0, 54.11, 54.12, 54.19, 54.3, 54.4, 54.51, 54.59, 54.61, 54.63, 54.64, 54.71, 54.72, 54.73, 54.74, 54.75, 54.92, 54.93 | 39502, 39503, 39540, 39541, 39545,  44188, 49020, 49040, 49060, 49062, 49460,  38570-38589, 43279-43289, 43324-43330, 44180-44185, 49000-49010, 49203-49220, 49320-49329, 49900-49999, 60650-60659 |

1. **Administrative codes used to define other variables**

| **Variable** | **ICD-9-CM diagnosis codes** | **CPT-4 codes** | **UB-92 revenue codes** | **Data sources** |
| --- | --- | --- | --- | --- |
| Outpatient dialysis encounters^a^ | V56.0, V56.8 | 90935, 90937, 90945, 90947 | 0820-0881 | Outpatient facility file and  Carrier claims |
| Surgery |  |  | 0360-0367, 0490-0499 | Inpatient or outpatient file for operating room |
| Outpatient clinic visits^b^ |  | 99201-99205, 99211-99215, 99241-99245, 99354-99357, 99387, 99397^c^; 99382-99386 |  | Carrier claims and outpatient files for patient consultation with a physician |
| Outpatient colonoscopy |  | 44388-44397 or 45355-45392 |  | Carrier claims and outpatient files |
| Treat and release ED visits |  |  | 0450-0459 | Outpatient file |
| Non-elective hospital admissions^d^ |  |  | 0450-0459 |  |

^a^Claims were merged by date to avoid double counting.

^b^Carrier claims that are nested within the dates of an inpatient admission were excluded. Carrier claims that matched with another eligible outpatient visit (colonoscopy, dialysis) were also excluded to avoid double counting.

^c^All patients.

^d^Defined as inpatient hospitalizations that came through the emergency department

**Table A: Prevalence of comorbidities, infections, healthcare exposures, and age groups; CDI incidence and population attributable risk percent (PAR%) of comorbidities, infections, healthcare exposures, and age groups.**

| **Characteristic** | **Prevalence of  exposure (n)** | **Prevalence of  exposure (%)** | **CDI Incidence  [per 100K population]** | **PAR%** |
| --- | --- | --- | --- | --- |
| **Comorbidities** |  |  |  |  |
| Arthritis | 43,630 | 3.0 | 1,336 | 3.3 |
| Congestive heart failure | 119,615 | 8.2 | 2,824 | 30.2 |
| Valvular disease | 80,301 | 5.5 | 1,889 | 11.3 |
| Chronic lung disease | 174,260 | 11.9 | 1,742 | 23.1 |
| Coagulopathy | 27,416 | 1.9 | 3,031 | 7.1 |
| Diabetes | 311,529 | 21.3 | 1,055 | 17.4 |
| Fluid and electrolyte disorder | 102,870 | 7.0 | 3,154 | 29.6 |
| Hypothyroidism | 177,681 | 12.1 | 1,179 | 11.6 |
| Hypertension | 793,466 | 54.1 | 971 | 60.6 |
| Liver disease | 9,303 | 0.6 | 2,376 | 1.7 |
| Obesity | 30,088 | 2.1 | 1,868 | 4.0 |
| Other neurological disorder | 98,884 | 6.7 | 2,528 | 21.3 |
| Paralysis | 17,501 | 1.2 | 4,011 | 6.3 |
| Pulmonary circulation disorder | 23,878 | 1.6 | 3,011 | 6.1 |
| Peripheral vascular disease | 133,829 | 9.1 | 1,965 | 20.7 |
| Renal failure | 88,239 | 6.0 | 2,757 | 21.1 |
| Lymphoma | 11,392 | 0.8 | 1,843 | 1.5 |
| Solid tumor | 115,603 | 7.9 | 1,130 | 6.5 |
| Metastatic cancer | 15,387 | 1.0 | 2,392 | 2.9 |
| Weight loss | 27,256 | 1.9 | 4,124 | 10.3 |
| Blood loss anemia | 11,194 | 0.8 | 3,305 | 3.2 |
| Deficiency anemia | 190,322 | 13.0 | 2,270 | 37.9 |
| Psychoses | 45,896 | 3.1 | 2,314 | 8.4 |
| Depression | 69,288 | 4.7 | 2,319 | 13.0 |
| Any comorbidity | 1,019,540 | 69.5 | 884 | 86.3 |
| No comorbidity | 446,387 | 30.5 | 88 | -37.8 |
| **Infections** |  |  |  |  |
| **Septicemia/endovascular infection** |  |  |  |  |
| ≤3 months | 12,121 | 0.8 | 19,710 | 24.8 |
| 3 - ≤6 months | 8,347 | 0.6 | 6,110 | 4.9 |
| 6 - ≤12 months | 13,544 | 0.9 | 4,031 | 4.9 |
| **Clinical sepsis** |  |  |  |  |
| ≤3 months | 8,725 | 0.6 | 16,825 | 15.1 |
| 3 - ≤6 months | 5,729 | 0.4 | 6,493 | 3.6 |
| 6 - ≤12 months | 9,206 | 0.6 | 4,236 | 3.5 |
| **Pneumonia** |  |  |  |  |
| ≤3 months | 37,517 | 2.6 | 9,230 | 35.2 |
| 3 - ≤6 months | 30,297 | 2.1 | 3,301 | 8.8 |
| 6 - ≤12 months | 48,846 | 3.3 | 2,397 | 9.4 |
| **Urinary tract infection** |  |  |  |  |
| ≤3 months | 117,127 | 8.0 | 3,632 | 40.5 |
| 3 - ≤6 months | 108,988 | 7.4 | 1,909 | 15.9 |
| 6 - ≤12 months | 173,858 | 11.9 | 1,513 | 18.3 |
| **Skin/soft tissue infection** |  |  |  |  |
| ≤3 months | 51,091 | 3.5 | 3,241 | 14.6 |
| 3 - ≤6 months | 47,973 | 3.3 | 1,857 | 6.4 |
| 6 - ≤12 months | 79,849 | 5.4 | 1,482 | 7.5 |
| **Surgical site infection** |  |  |  |  |
| ≤3 months | 4,218 | 0.3 | 11,617 | 4.9 |
| 3 - ≤6 months | 3,648 | 0.2 | 4,441 | 1.5 |
| 6 - ≤12 months | 6,226 | 0.4 | 3,036 | 1.6 |
| **Bone/joint infection** |  |  |  |  |
| ≤3 months | 4,343 | 0.3 | 8,312 | 3.6 |
| 3 - ≤6 months | 3,793 | 0.3 | 4,086 | 1.4 |
| 6 - ≤12 months | 6,076 | 0.4 | 2,518 | 1.2 |
| **Organ abscess/meningitis** |  |  |  |  |
| ≤3 months | 1,621 | 0.1 | 6,539 | 1.0 |
| 3 - ≤6 months | 1,431 | 0.1 | 1,607 | 0.1 |
| 6 - ≤12 months | 2,639 | 0.2 | 1,288 | 0.2 |
| **Female pelvic infection** |  |  |  |  |
| ≤3 months | 7,360 | 0.5 | 1,467 | 0.7 |
| 3 - ≤6 months | 7,258 | 0.5 | 937 | 0.2 |
| 6 - ≤12 months | 13,795 | 0.9 | 739 | 0.1 |
| **Gastroenteritis** |  |  |  |  |
| ≤3 months | 2,674 | 0.2 | 13,239 | 3.6 |
| 3 - ≤6 months | 2,190 | 0.1 | 5,982 | 1.2 |
| 6 - ≤12 months | 4,195 | 0.3 | 3,361 | 1.2 |
| **Intraabdominal abscess** |  |  |  |  |
| ≤3 months | 47,932 | 3.3 | 3,530 | 15.2 |
| 3 - ≤6 months | 45,396 | 3.1 | 1,445 | 4.0 |
| 6 - ≤12 months | 82,543 | 5.6 | 1,061 | 3.9 |
| **Upper respiratory tract infection** |  |  |  |  |
| ≤3 months | 106,862 | 7.3 | 1,232 | 7.2 |
| 3 - ≤6 months | 103,228 | 7.0 | 854 | 2.5 |
| 6 - ≤12 months | 179,971 | 12.3 | 786 | 3.2 |
| **Tonsillitis** |  |  |  |  |
| ≤3 months | 7,155 | 0.5 | 1,831 | 0.9 |
| 3 - ≤6 months | 6,942 | 0.5 | 1,253 | 0.5 |
| 6 - ≤12 months | 12,609 | 0.9 | 1,007 | 0.5 |
| **Otitis media** |  |  |  |  |
| ≤3 months | 12,441 | 0.8 | 836 | 0.3 |
| 3 - ≤6 months | 12,272 | 0.8 | 904 | 0.3 |
| 6 - ≤12 months | 22,397 | 0.8 | 866 | 0.5 |
| **Oral abscess** |  |  |  |  |
| ≤3 months | 2,332 | 0.2 | 1,501 | 0.2 |
| 3 - ≤6 months | 2,213 | 0.2 | 813 | 0.04 |
| 6 - ≤12 months | 4,029 | 0.3 | 968 | 0.1 |
| **Viral infection** |  |  |  |  |
| ≤3 months | 15,632 | 1.1 | 2,629 | 3.3 |
| 3 - ≤6 months | 13,844 | 0.9 | 1,264 | 0.9 |
| 6 - ≤12 months | 25,735 | 0.2 | 1,107 | 1.3 |
| **Any infection** |  |  |  |  |
| ≤3 months | 328,815 | 22.4 | 2,292 | 74.4 |
| 3 - ≤6 months | 312,007 | 21.3 | 1,337 | 29.4 |
| 6 - ≤12 months | 480,429 | 32.8 | 1,056 | 31.5 |
| **No infection** |  |  |  |  |
| ≤3 months | 1,137,112 | 77.6 | 164 | -257.4 |
| 3 - ≤6 months | 959,670 | 65.5 | 135 | -149.8 |
| 6 - ≤12 months | 740,384 | 50.5 | 108 | -84.9 |
| **Healthcare Exposures** |  |  |  |  |
| **Any hospitalization in past 12 months** |  |  |  |  |
| 1 | 186,540 | 12.7 | 1,561 | 20.9 |
| >=1 | 279,797 | 19.1 | 2,559 | 70.5 |
| >=2 | 93,257 | 6.4 | 4,555 | 41.5 |
| **Non-emergent hospitalization** |  |  |  |  |
| ≤3 months | 29,905 | 2.0 | 2,989 | 7.6 |
| 3 - ≤6 months | 29,054 | 2.0 | 1,958 | 4.2 |
| 6 - ≤12 months | 54,184 | 3.7 | 1,200 | 3.3 |
| **Emergent Hospitalization** |  |  |  |  |
| ≤3 months | 73,779 | 5.0 | 6,514 | 48.5 |
| 3 - ≤6 months | 62,788 | 4.3 | 2,891 | 15.7 |
| 6 - ≤12 months | 105,041 | 7.2 | 2,073 | 17.2 |
| **Surgery** |  |  |  |  |
| ≤3 months | 32,599 | 2.2 | 6,172 | 19.6 |
| 3 - ≤6 months | 29,227 | 2.0 | 1,896 | 4.0 |
| 6 - ≤12 months | 54,093 | 3.7 | 1,135 | 3.0 |
| **Emergency department visit without hospitalization** |  |  |  |  |
| ≤3 months | 89,635 | 6.1 | 2,840 | 22.3 |
| 3 - ≤6 months | 84,876 | 5.8 | 1,314 | 6.4 |
| 6 - ≤12 months | 141,964 | 9.7 | 1,075 | 7.3 |
| **Invasive procedure** |  |  |  |  |
| ≤3 months | 90,569 | 6.2 | 4,432 | 38.9 |
| 3 - ≤6 months | 84,369 | 5.8 | 1,701 | 10.1 |
| 6 - ≤12 months | 150,124 | 10.2 | 1,192 | 9.8 |
| **Outpatient invasive procedure** |  |  |  |  |
| ≤3 months | 55,097 | 3.8 | 1,361 | 4.4 |
| 3 - ≤6 months | 53,314 | 3.6 | 1,191 | 3.2 |
| 6 - ≤12 months | 96,653 | 6.6 | 955 | 3.5 |
| **Outpatient dialysis** |  |  |  |  |
| ≤3 months | 1,990 | 0.1 | 5,276 | 1.0 |
| 3 - ≤6 months | 3,322 | 0.2 | 4,696 | 1.4 |
| 6 - ≤12 months | 5,756 | 0.4 | 4,500 | 2.4 |
| **Not a nursing home resident and no skilled nursing exposures (no NH/no SNF)** |  |  |  |  |
| ≤3 months | 1,270,210 | 86.6 | 424 | -218.3 |
| 3 - ≤6 months | 1,281,815 | 87.4 | 538 | -111.3 |
| 6 - ≤12 months | 1,283,419 | 87.5 | 561 | -87.2 |
| **Nursing home resident with no skilled nursing exposure (yes NH/no SNF)** |  |  |  |  |
| ≤3 months | 65,354 | 4.5 | 2,442 | 13.1 |
| 3 - ≤6 months | 59,514 | 4.1 | 2,460 | 12.0 |
| 6 - ≤12 months | 35,675 | 2.4 | 3,221 | 10.0 |
| **Not a nursing home resident with a skilled nursing exposure (no NH/yes SNF)** |  |  |  |  |
| ≤3 months | 21,605 | 1.5 | 7,762 | 16.6 |
| 3 - ≤6 months | 17,977 | 1.2 | 3,432 | 5.4 |
| 6 - ≤12 months | 24,261 | 1.7 | 2,568 | 5.1 |
| **Nursing home resident with a skilled nursing exposure (yes NH/yes SNF)** |  |  |  |  |
| ≤3 months | 18,573 | 1.3 | 3,995 | 6.7 |
| 3 - ≤6 months | 16,436 | 1.1 | 2,598 | 3.5 |
| 6 - ≤12 months | 32,387 | 2.2 | 1,328 | 2.4 |
| **Age categories** |  |  |  |  |
| 66-70 | 410,245 | 28.0 | 321 | -19.4 |
| 71-75 | 331,353 | 22.6 | 425 | -9.8 |
| 76-80 | 283,348 | 19.3 | 619 | -0.8 |
| 81-85 | 227,481 | 15.5 | 915 | 7.9 |
| ≥86 | 213,500 | 14.6 | 1,330 | 18.3 |

**Table B: Prevalence of comorbidities, infections within 3 months of CDI, and healthcare exposures within 3 months of CDI and CDI incidence and population attributable risk percent (PAR%) of comorbidities, infections within 3 months of CDI, and healthcare exposures within 3 months of CDI stratified by age.**

| **Characteristic** | **Prevalence of  exposure (n)** | **Prevalence of  exposure (%)** | **CDI Incidence  [per 100K population]** | **PAR%** |
| --- | --- | --- | --- | --- |
| **Comorbidities** |  |  |  |  |
| **Arthritis** |  |  |  |  |
| 66-70 | 9,344 | 0.6 | 824 | 0.2 |
| 71-75 | 9,873 | 0.7 | 1,023 | 0.4 |
| 76-80 | 9,548 | 0.7 | 1,309 | 0.7 |
| 81-85 | 7,383 | 0.5 | 1,950 | 1.0 |
| ≥86 | 5,358 | 0.4 | 2,538 | 1.1 |
| **Congestive heart failure** |  |  |  |  |
| 66-70 | 13,625 | 0.9 | 2,613 | 2.9 |
| 71-75 | 17,432 | 1.2 | 2,386 | 3.3 |
| 76-80 | 22,672 | 1.5 | 2,673 | 5.0 |
| 81-85 | 26,092 | 1.8 | 3,101 | 7.0 |
| ≥86 | 36,063 | 2.5 | 3,303 | 10.5 |
| **Valvular disease** |  |  |  |  |
| 66-70 | 10,717 | 0.7 | 1,362 | 0.8 |
| 71-75 | 13,754 | 0.9 | 1,498 | 1.3 |
| 76-80 | 17,615 | 1.2 | 1,641 | 1.9 |
| 81-85 | 17,913 | 1.2 | 2,032 | 2.7 |
| ≥86 | 17,909 | 1.2 | 2,859 | 4.3 |
| **Chronic lung disease** |  |  |  |  |
| 66-70 | 35,909 | 2.4 | 1,376 | 2.9 |
| 71-75 | 36,904 | 2.5 | 1,314 | 2.7 |
| 76-80 | 36,141 | 2.5 | 1,594 | 3.8 |
| 81-85 | 30,092 | 2.1 | 2,117 | 4.8 |
| ≥86 | 26,503 | 1.8 | 3,181 | 7.3 |
| **Coagulopathy** |  |  |  |  |
| 66-70 | 4,277 | 0.3 | 2,736 | 1.0 |
| 71-75 | 5,113 | 0.3 | 2,797 | 1.2 |
| 76-80 | 5,832 | 0.4 | 3,069 | 1.5 |
| 81-85 | 5,683 | 0.4 | 3,203 | 1.6 |
| ≥86 | 5,514 | 0.4 | 3,808 | 1.9 |
| **Diabetes** |  |  |  |  |
| 66-70 | 72,868 | 5.0 | 788 | 1.2 |
| 71-75 | 71,467 | 4.9 | 856 | 1.7 |
| 76-80 | 63,635 | 4.3 | 1,070 | 3.0 |
| 81-85 | 49,070 | 3.3 | 1,390 | 4.0 |
| ≥86 | 36,646 | 2.5 | 2,011 | 5.5 |
| **Fluid and electrolyte disorder** |  |  |  |  |
| 66-70 | 14,819 | 1.0 | 3,043 | 3.8 |
| 71-75 | 17,062 | 1.2 | 2,766 | 3.9 |
| 76-80 | 19,961 | 1.4 | 2,921 | 4.9 |
| 81-85 | 21,191 | 1.4 | 3,468 | 6.5 |
| ≥86 | 26,144 | 1.8 | 3,840 | 9.1 |
| **Hypothyroidism** |  |  |  |  |
| 66-70 | 31,432 | 2.1 | 703 | 0.2 |
| 71-75 | 33,917 | 2.3 | 820 | 0.7 |
| 76-80 | 35,775 | 2.4 | 953 | 1.2 |
| 81-85 | 33,589 | 2.3 | 1,486 | 3.1 |
| ≥86 | 35,806 | 2.4 | 2,111 | 5.7 |
| **Hypertension** |  |  |  |  |
| 66-70 | 160,673 | 11.0 | 614 | -0.5 |
| 71-75 | 165,437 | 11.3 | 681 | 0.8 |
| 76-80 | 160,569 | 10.9 | 907 | 5.1 |
| 81-85 | 139,028 | 9.5 | 1,269 | 10.3 |
| ≥86 | 130,337 | 8.9 | 1,819 | 17.9 |
| **Liver disease** |  |  |  |  |
| 66-70 | 3,024 | 0.2 | 2,083 | 0.5 |
| 71-75 | 2,274 | 0.2 | 1,847 | 0.3 |
| 76-80 | 1,694 | 0.1 | 2,834 | 0.4 |
| 81-85 | 1,002 | 0.1 | 3,892 | 0.3 |
| ≥86 | 556 | 0.0 | 5,216 | 0.3 |
| **Obesity** |  |  |  |  |
| 66-70 | 10,260 | 0.7 | 1,491 | 0.9 |
| 71-75 | 7,787 | 0.5 | 1,901 | 1.0 |
| 76-80 | 5,195 | 0.4 | 2,214 | 0.9 |
| 81-85 | 2,881 | 0.2 | 3,124 | 0.8 |
| ≥86 | 1,391 | 0.1 | 4,026 | 0.5 |
| **Other neurological disorder** |  |  |  |  |
| 66-70 | 11,380 | 0.8 | 2,364 | 2.1 |
| 71-75 | 14,157 | 1.0 | 2,246 | 2.4 |
| 76-80 | 18,880 | 1.3 | 2,320 | 3.4 |
| 81-85 | 22,653 | 1.5 | 2,649 | 4.9 |
| ≥86 | 28,965 | 2.0 | 3,021 | 7.5 |
| **Paralysis** |  |  |  |  |
| 66-70 | 2,845 | 0.2 | 4,183 | 1.1 |
| 71-75 | 3,120 | 0.2 | 3,622 | 1.0 |
| 76-80 | 3,526 | 0.2 | 4,169 | 1.3 |
| 81-85 | 3,559 | 0.2 | 4,130 | 1.3 |
| ≥86 | 3,678 | 0.3 | 4,785 | 1.6 |
| **Pulmonary circulation disorder** |  |  |  |  |
| 66-70 | 3,532 | 0.2 | 3,001 | 0.9 |
| 71-75 | 4,398 | 0.3 | 2,842 | 1.0 |
| 76-80 | 5,003 | 0.3 | 2,858 | 1.2 |
| 81-85 | 4,990 | 0.3 | 3,367 | 1.5 |
| ≥86 | 5,072 | 0.3 | 3,490 | 1.5 |
| **Peripheral vascular disease** |  |  |  |  |
| 66-70 | 16,082 | 1.1 | 1,766 | 1.9 |
| 71-75 | 21,045 | 1.4 | 1,668 | 2.3 |
| 76-80 | 26,430 | 1.8 | 1,892 | 3.6 |
| 81-85 | 29,110 | 2.0 | 2,016 | 4.3 |
| ≥86 | 37,262 | 2.5 | 2,437 | 7.3 |
| **Renal failure** |  |  |  |  |
| 66-70 | 12,956 | 0.9 | 2,732 | 2.9 |
| 71-75 | 15,787 | 1.1 | 2,496 | 3.1 |
| 76-80 | 18,236 | 1.2 | 2,522 | 3.7 |
| 81-85 | 18,473 | 1.3 | 2,945 | 4.6 |
| ≥86 | 19,513 | 1.3 | 3,490 | 6.0 |
| **Lymphoma** |  |  |  |  |
| 66-70 | 2,305 | 0.2 | 1,735 | 0.3 |
| 71-75 | 2,459 | 0.2 | 1,952 | 0.3 |
| 76-80 | 2,553 | 0.2 | 1,958 | 0.4 |
| 81-85 | 2,178 | 0.1 | 1,882 | 0.3 |
| ≥86 | 1,450 | 0.1 | 2,138 | 0.2 |
| **Solid tumor** |  |  |  |  |
| 66-70 | 24,151 | 1.6 | 907 | 0.7 |
| 71-75 | 26,285 | 1.8 | 856 | 0.6 |
| 76-80 | 25,253 | 1.7 | 1,164 | 1.4 |
| 81-85 | 20,300 | 1.4 | 1,419 | 1.7 |
| ≥86 | 14,540 | 1.0 | 1,926 | 2.0 |
| **Metastatic cancer** |  |  |  |  |
| 66-70 | 3,591 | 0.2 | 2,256 | 0.6 |
| 71-75 | 3,605 | 0.2 | 2,219 | 0.6 |
| 76-80 | 3,228 | 0.2 | 2,912 | 0.8 |
| 81-85 | 2,498 | 0.2 | 2,802 | 0.6 |
| ≥86 | 1,671 | 0.1 | 2,573 | 0.3 |
| **Weight loss** |  |  |  |  |
| 66-70 | 3,493 | 0.2 | 4,695 | 1.5 |
| 71-75 | 4,070 | 0.3 | 4,128 | 1.5 |
| 76-80 | 5,425 | 0.4 | 4,111 | 2.0 |
| 81-85 | 5,981 | 0.4 | 3,979 | 2.1 |
| ≥86 | 7,430 | 0.5 | 4,455 | 3.0 |
| **Blood loss anemia** |  |  |  |  |
| 66-70 | 1,534 | 0.1 | 2,999 | 0.4 |
| 71-75 | 1,925 | 0.1 | 3,688 | 0.6 |
| 76-80 | 2,239 | 0.2 | 2,724 | 0.5 |
| 81-85 | 2,425 | 0.2 | 3,134 | 0.6 |
| ≥86 | 2,690 | 0.2 | 4,312 | 1.1 |
| **Deficiency anemia** |  |  |  |  |
| 66-70 | 27,503 | 1.9 | 2,003 | 4.1 |
| 71-75 | 32,871 | 2.2 | 1,901 | 4.5 |
| 76-80 | 38,296 | 2.6 | 2,094 | 6.1 |
| 81-85 | 39,635 | 2.7 | 2,430 | 7.8 |
| ≥86 | 45,356 | 3.1 | 3,040 | 11.9 |
| **Psychoses** |  |  |  |  |
| 66-70 | 10,182 | 0.7 | 1,424 | 0.9 |
| 71-75 | 8,596 | 0.6 | 1,920 | 1.2 |
| 76-80 | 8,079 | 0.6 | 2,401 | 1.5 |
| 81-85 | 7,718 | 0.5 | 3,330 | 2.2 |
| ≥86 | 8,490 | 0.6 | 3,545 | 2.6 |
| **Depression** |  |  |  |  |
| 66-70 | 12,828 | 0.9 | 1,777 | 1.6 |
| 71-75 | 12,095 | 0.8 | 1,943 | 1.7 |
| 76-80 | 12,827 | 0.9 | 2,237 | 2.2 |
| 81-85 | 12,899 | 0.9 | 2,876 | 3.1 |
| ≥86 | 15,264 | 1.0 | 3,184 | 4.2 |
| **Infections** |  |  |  |  |
| **Septicemia/endovascular infection** |  |  |  |  |
| 66-70 | 1,838 | 0.1 | 21,001 | 4.0 |
| 71-75 | 2,022 | 0.1 | 20,574 | 4.3 |
| 76-80 | 2,358 | 0.2 | 19,423 | 4.7 |
| 81-85 | 2,458 | 0.2 | 22,457 | 5.7 |
| ≥86 | 2,986 | 0.2 | 22,036 | 6.8 |
| **Pneumonia** |  |  |  |  |
| 66-70 | 5,375 | 0.4 | 8,540 | 4.5 |
| 71-75 | 6,051 | 0.4 | 8,263 | 4.9 |
| 76-80 | 7,120 | 0.5 | 8,539 | 6.0 |
| 81-85 | 7,517 | 0.5 | 10,696 | 8.1 |
| ≥86 | 10,084 | 0.7 | 10,918 | 11.1 |
| **Urinary tract infection** |  |  |  |  |
| 66-70 | 18,360 | 1.3 | 2,658 | 4.0 |
| 71-75 | 20,585 | 1.4 | 2,822 | 4.8 |
| 76-80 | 22,691 | 1.5 | 3,601 | 7.3 |
| 81-85 | 23,162 | 1.6 | 4,758 | 10.3 |
| ≥86 | 27,866 | 1.9 | 5,598 | 15.0 |
| **Skin/soft tissue infection** |  |  |  |  |
| 66-70 | 8,856 | 0.6 | 2,721 | 2.0 |
| 71-75 | 9,216 | 0.6 | 2,897 | 2.2 |
| 76-80 | 9,849 | 0.7 | 3,148 | 2.6 |
| 81-85 | 9,775 | 0.7 | 3,765 | 3.3 |
| ≥86 | 11,194 | 0.8 | 4,413 | 4.5 |
| **Intraabdominal abscess** |  |  |  |  |
| 66-70 | 10,703 | 0.7 | 2,681 | 2.3 |
| 71-75 | 10,844 | 0.7 | 2,905 | 2.6 |
| 76-80 | 10,034 | 0.7 | 3,687 | 3.3 |
| 81-85 | 7,859 | 0.5 | 5,153 | 3.8 |
| ≥86 | 5,957 | 0.4 | 7,605 | 4.4 |
| **Healthcare Exposures** |  |  |  |  |
| **Surgery** |  |  |  |  |
| 66-70 | 7,470 | 0.5 | 4,337 | 3.0 |
| 71-75 | 7,237 | 0.5 | 5,278 | 3.6 |
| 76-80 | 6,675 | 0.5 | 6,502 | 4.2 |
| 81-85 | 5,306 | 0.4 | 8,387 | 4.4 |
| ≥86 | 4,086 | 0.3 | 10,450 | 4.3 |
| **Invasive procedure** |  |  |  |  |
| 66-70 | 20,533 | 1.4 | 3,258 | 5.8 |
| 71-75 | 20,242 | 1.4 | 3,498 | 6.2 |
| 76-80 | 18,840 | 1.3 | 4,448 | 7.7 |
| 81-85 | 14,899 | 1.0 | 5,866 | 8.4 |
| ≥86 | 11,044 | 0.8 | 8,376 | 9.2 |
| **Outpatient invasive procedure** |  |  |  |  |
| 66-70 | 13,367 | 0.9 | 1,100 | 0.7 |
| 71-75 | 12,982 | 0.9 | 1,132 | 0.7 |
| 76-80 | 11,512 | 0.8 | 1,390 | 0.9 |
| 81-85 | 8,615 | 0.6 | 1,950 | 1.2 |
| ≥86 | 5,479 | 0.4 | 2,336 | 1.0 |
| **Dialysis** |  |  |  |  |
| 66-70 | 472 | 0.0 | 4,661 | 0.2 |
| 71-75 | 436 | 0.0 | 6,422 | 0.3 |
| 76-80 | 455 | 0.0 | 5,275 | 0.2 |
| 81-85 | 334 | 0.0 | 6,587 | 0.2 |
| ≥86 | 174 | 0.0 | 5,172 | 0.1 |
| **Not a nursing home resident and no skilled nursing exposures (no NH/no SNF)** |  |  |  |  |
| 66-70 | 322,800 | 22.0 | 300 | -15.0 |
| 71-75 | 283,773 | 19.3 | 346 | -11.0 |
| 76-80 | 243,274 | 16.6 | 454 | -5.8 |
| 81-85 | 187,006 | 12.7 | 606 | -0.8 |
| ≥86 | 145,575 | 9.9 | 822 | 3.1 |
| **Nursing home resident with no skilled nursing exposure (yes NH/no SNF)** |  |  |  |  |
| 66-70 | 3,949 | 0.3 | 2,963 | 1.0 |
| 71-75 | 5,315 | 0.4 | 2,860 | 1.3 |
| 76-80 | 8,941 | 0.6 | 2,393 | 1.7 |
| 81-85 | 14,589 | 1.0 | 2,584 | 3.0 |
| ≥86 | 31,298 | 2.1 | 2,352 | 5.8 |
| **Not a nursing home resident with a skilled nursing exposure (no NH/yes SNF)** |  |  |  |  |
| 66-70 | 1,836 | 0.1 | 9,423 | 1.7 |
| 71-75 | 2,676 | 0.2 | 7,287 | 1.9 |
| 76-80 | 4,155 | 0.3 | 8,039 | 3.3 |
| 81-85 | 5,211 | 0.4 | 7,810 | 4.0 |
| ≥86 | 7,237 | 0.5 | 7,849 | 5.6 |
| **Nursing home resident with a skilled nursing exposure (yes NH/yes SNF)** |  |  |  |  |
| 66-70 | 1,251 | 0.1 | 4,636 | 0.5 |
| 71-75 | 1,729 | 0.1 | 4,627 | 0.7 |
| 76-80 | 2,726 | 0.2 | 3,705 | 0.9 |
| 81-85 | 4,312 | 0.3 | 3,827 | 1.5 |
| ≥86 | 8,173 | 0.6 | 4,136 | 3.1 |
| 76-80 | 9,849 | 0.7 | 3,148 | 2.6 |
| 81-85 | 9,775 | 0.7 | 3,765 | 3.3 |
| ≥86 | 11,194 | 0.8 | 4,413 | 4.5 |

**Table C: Prevalence of comorbidities, infections within 3 months of CDI, and healthcare exposures within 3 months of CDI and CDI incidence and population attributable risk percent (PAR%) of comorbidities, infections, healthcare exposures, and age stratified by hospital exposure within 3 months of CDI.**

| **Characteristic** | **Prevalence of  exposure (n)** | **Prevalence of  exposure (%)** | **CDI Incidence  [per 100K population]** | **PAR%** |
| --- | --- | --- | --- | --- |
| **Comorbidities** |  |  |  |  |
| **Arthritis** |  |  |  |  |
| Emergent hospitalization | 4,104 | 0.3 | 7,554 | 3.0 |
| Non-emergent hospitalization | 1,527 | 0.1 | 4,650 | 0.7 |
| No hospitalization | 37,999 | 2.6 | 532 | -0.5 |
| **Congestive heart failure** |  |  |  |  |
| Emergent hospitalization | 28,107 | 1.9 | 7,884 | 22.1 |
| Non-emergent hospitalization | 5,514 | 0.4 | 5,767 | 3.0 |
| No hospitalization | 85,994 | 5.9 | 981 | 3.3 |
| **Valvular disease** |  |  |  |  |
| Emergent hospitalization | 14,043 | 1.0 | 7,185 | 9.9 |
| Non-emergent hospitalization | 3,825 | 0.3 | 4,183 | 1.4 |
| No hospitalization | 62,433 | 4.3 | 557 | -0.6 |
| **Chronic lung disease** |  |  |  |  |
| Emergent hospitalization | 27,042 | 1.8 | 6,915 | 18.4 |
| Non-emergent hospitalization | 7,220 | 0.5 | 4,335 | 2.9 |
| No hospitalization | 139,998 | 9.6 | 609 | -0.5 |
| **Coagulopathy** |  |  |  |  |
| Emergent hospitalization | 6,002 | 0.4 | 9,147 | 5.5 |
| Non-emergent hospitalization | 1,585 | 0.1 | 5,931 | 0.9 |
| No hospitalization | 19,829 | 1.4 | 948 | 0.7 |
| **Diabetes** |  |  |  |  |
| Emergent hospitalization | 27,371 | 1.9 | 6,814 | 18.3 |
| Non-emergent hospitalization | 8,149 | 0.6 | 4,221 | 3.1 |
| No hospitalization | 54,028 | 18.8 | 390 | -9.1 |
| **Fluid and electrolyte disorder** |  |  |  |  |
| Emergent hospitalization | 27,014 | 1.8 | 7,748 | 20.8 |
| Non-emergent hospitalization | 5,272 | 0.4 | 5,596 | 2.8 |
| No hospitalization | 70,584 | 4.8 | 1,214 | 4.5 |
| **Hypothyroidism** |  |  |  |  |
| Emergent hospitalization | 16,770 | 1.1 | 6,798 | 11.1 |
| Non-emergent hospitalization | 5,497 | 0.4 | 3,711 | 1.8 |
| No hospitalization | 155,414 | 10.6 | 483 | -2.9 |
| **Hypertension** |  |  |  |  |
| Emergent hospitalization | 64,185 | 4.4 | 6,614 | 42.6 |
| Non-emergent hospitalization | 21,847 | 1.5 | 3,694 | 7.2 |
| No hospitalization | 707,434 | 48.3 | 375 | -38.7 |
| **Liver disease** |  |  |  |  |
| Emergent hospitalization | 1,663 | 0.1 | 8,539 | 1.4 |
| Non-emergent hospitalization | 444 | 0.0 | 4,054 | 0.2 |
| No hospitalization | 7,196 | 0.5 | 848 | 0.2 |
| **Obesity** |  |  |  |  |
| Emergent hospitalization | 5,356 | 0.4 | 6,385 | 3.3 |
| Non-emergent hospitalization | 2,370 | 0.2 | 3,671 | 0.8 |
| No hospitalization | 22,362 | 1.5 | 595 | -0.1 |
| **Other neurological disorder** |  |  |  |  |
| Emergent hospitalization | 18,761 | 1.3 | 8,246 | 15.4 |
| Non-emergent hospitalization | 3,101 | 0.2 | 5,256 | 1.5 |
| No hospitalization | 77,022 | 5.3 | 1,026 | 3.3 |
| **Paralysis** |  |  |  |  |
| Emergent hospitalization | 4,993 | 0.3 | 9,173 | 4.5 |
| Non-emergent hospitalization | 717 | 0.0 | 9,205 | 0.7 |
| No hospitalization | 11,791 | 0.8 | 1,510 | 1.1 |
| **Pulmonary circulation disorder** |  |  |  |  |
| Emergent hospitalization | 6,686 | 0.5 | 7,688 | 5.0 |
| Non-emergent hospitalization | 1,342 | 0.1 | 4,993 | 0.6 |
| No hospitalization | 15,850 | 1.1 | 871 | 0.4 |
| **Peripheral vascular disease** |  |  |  |  |
| Emergent hospitalization | 18,738 | 1.3 | 8,032 | 14.9 |
| Non-emergent hospitalization | 5,340 | 0.4 | 4,981 | 2.5 |
| No hospitalization | 109,751 | 7.5 | 783 | 1.8 |
| **Renal failure** |  |  |  |  |
| Emergent hospitalization | 18,448 | 1.3 | 8,603 | 15.8 |
| Non-emergent hospitalization | 4,003 | 0.3 | 6,195 | 2.4 |
| No hospitalization | 65,788 | 4.5 | 909 | 2.0 |
| **Lymphoma** |  |  |  |  |
| Emergent hospitalization | 1,376 | 0.1 | 7,849 | 1.1 |
| Non-emergent hospitalization | 536 | 0.0 | 5,784 | 0.3 |
| No hospitalization | 9,480 | 0.6 | 749 | 0.1 |
| **Solid tumor** |  |  |  |  |
| Emergent hospitalization | 10,712 | 0.7 | 6,833 | 7.1 |
| Non-emergent hospitalization | 5,408 | 0.4 | 3,439 | 1.6 |
| No hospitalization | 99,483 | 6.8 | 390 | -2.9 |
| **Metastatic cancer** |  |  |  |  |
| Emergent hospitalization | 3,672 | 0.3 | 6,073 | 2.1 |
| Non-emergent hospitalization | 1,559 | 0.1 | 3,784 | 0.5 |
| No hospitalization | 10,156 | 0.7 | 847 | 0.2 |
| **Weight loss** |  |  |  |  |
| Emergent hospitalization | 6,682 | 0.5 | 10,730 | 7.2 |
| Non-emergent hospitalization | 1,397 | 0.1 | 8,304 | 1.1 |
| No hospitalization | 19,177 | 1.3 | 1,517 | 1.8 |
| **Blood loss anemia** |  |  |  |  |
| Emergent hospitalization | 2,900 | 0.2 | 8,828 | 2.5 |
| Non-emergent hospitalization | 803 | 0.1 | 5,355 | 0.4 |
| No hospitalization | 7,491 | 0.5 | 948 | 0.2 |
| **Deficiency anemia** |  |  |  |  |
| Emergent hospitalization | 31,081 | 2.1 | 8,352 | 26.0 |
| Non-emergent hospitalization | 8,220 | 0.6 | 5,158 | 4.0 |
| No hospitalization | 151,021 | 10.3 | 861 | 3.9 |
| **Psychoses** |  |  |  |  |
| Emergent hospitalization | 7,490 | 0.5 | 8,011 | 5.9 |
| Non-emergent hospitalization | 1,527 | 0.1 | 4,191 | 0.6 |
| No hospitalization | 36,879 | 2.5 | 1,079 | 1.8 |
| **Depression** |  |  |  |  |
| Emergent hospitalization | 12,137 | 0.8 | 7,654 | 9.1 |
| Non-emergent hospitalization | 3,123 | 0.2 | 4,131 | 1.2 |
| No hospitalization | 54,028 | 3.7 | 1,016 | 2.2 |
| **Infections** |  |  |  |  |
| **Septicemia/endovascular infection** |  |  |  |  |
| Emergent hospitalization | 8,994 | 0.6 | 19,335 | 18.0 |
| Non-emergent hospitalization | 919 | 0.1 | 27,530 | 2.6 |
| No hospitalization | 2,208 | 0.2 | 21,649 | 4.9 |
| **Pneumonia** |  |  |  |  |
| Emergent hospitalization | 20,090 | 1.4 | 12,111 | 24.9 |
| Non-emergent hospitalization | 2,749 | 0.2 | 12,259 | 3.4 |
| No hospitalization | 14,678 | 1.0 | 4,783 | 6.5 |
| **Urinary tract infection** |  |  |  |  |
| Emergent hospitalization | 26,176 | 1.8 | 10,697 | 28.5 |
| Non-emergent hospitalization | 5,311 | 0.4 | 8,040 | 4.2 |
| No hospitalization | 85,640 | 5.8 | 1,543 | 8.7 |
| **Skin/soft tissue infection** |  |  |  |  |
| Emergent hospitalization | 9,072 | 0.6 | 10,880 | 9.9 |
| Non-emergent hospitalization | 2,656 | 0.2 | 8,735 | 2.3 |
| No hospitalization | 39,363 | 2.7 | 1,171 | 2.3 |
| **Intraabdominal abscess** |  |  |  |  |
| Emergent hospitalization | 10,957 | 0.7 | 10,176 | 11.2 |
| Non-emergent hospitalization | 2,838 | 0.2 | 7,435 | 2.1 |
| No hospitalization | 34,137 | 2.3 | 1,476 | 3.1 |
| **Healthcare Exposures** |  |  |  |  |
| **Surgery** |  |  |  |  |
| Emergent hospitalization | 11,656 | 0.8 | 10,681 | 12.5 |
| Non-emergent hospitalization | 13,881 | 0.9 | 3,227 | 3.9 |
| No hospitalization | 7,062 | 0.5 | 4,517 | 2.9 |
| **Invasive procedure** |  |  |  |  |
| Emergent hospitalization | 23,077 | 1.6 | 10,920 | 25.6 |
| Non-emergent hospitalization | 17,764 | 1.2 | 3,698 | 5.8 |
| No hospitalization | 49,728 | 3.4 | 1,683 | 5.7 |
| **Outpatient invasive procedure** |  |  |  |  |
| Emergent hospitalization | 5,221 | 0.4 | 7,029 | 3.6 |
| Non-emergent hospitalization | 2,686 | 0.2 | 3,909 | 0.9 |
| No hospitalization | 47,190 | 3.2 | 589 | -0.3 |
| **Dialysis** |  |  |  |  |
| Emergent hospitalization | 689 | 0.0 | 9,289 | 0.6 |
| Non-emergent hospitalization | 113 | 0.0 | 5,310 | 0.1 |
| No hospitalization | 1,188 | 0.1 | 2,946 | 0.3 |
| **Not a nursing home resident and no skilled nursing exposures (no NH/no SNF)** |  |  |  |  |
| Emergent hospitalization | 49,354 | 3.4 | 4,739 | 22.3 |
| Non-emergent hospitalization | 22,197 | 1.5 | 2,861 | 5.3 |
| No hospitalization | 1,198,659 | 81.7 | 201 | -306.4 |
| **Nursing home resident with no skilled nursing exposures (yes NH/no SNF)** |  |  |  |  |
| Emergent hospitalization | 6,480 | 0.4 | 10,093 | 6.5 |
| Non-emergent hospitalization | 800 | 0.1 | 8,500 | 0.7 |
| No hospitalization | 58,074 | 4.0 | 1,505 | 5.6 |
| **Not a nursing home resident with a skilled nursing exposure (no NH/yes SNF)** |  |  |  |  |
| Emergent hospitalization | 11,335 | 0.8 | 11,442 | 13.1 |
| Non-emergent hospitalization | 2,658 | 0.2 | 7,186 | 1.9 |
| No hospitalization | 7,612 | 0.5 | 2,483 | 1.5 |
| **Nursing home resident with a skilled nursing exposure (yes NH/yes SNF)** |  |  |  |  |
| Emergent hospitalization | 6,610 | 0.5 | 7,806 | 5.1 |
| Non-emergent hospitalization | 875 | 0.1 | 5,029 | 0.4 |
| No hospitalization | 11,088 | 0.8 | 1,641 | 1.2 |
| **Age categories** |  |  |  |  |
| **66-70** |  |  |  |  |
| Emergent hospitalization | 10,196 | 0.7 | 5,914 | 5.8 |
| Non-emergent hospitalization | 6,997 | 0.5 | 2,372 | 1.3 |
| No hospitalization | 313,441 | 21.4 | 175 | -19.7 |
| **71-75** |  |  |  |  |
| Emergent hospitalization | 11,761 | 0.8 | 5,765 | 6.5 |
| Non-emergent hospitalization | 6,936 | 0.5 | 2,682 | 1.5 |
| No hospitalization | 275,645 | 18.8 | 198 | -16.0 |
| **76-80** |  |  |  |  |
| Emergent hospitalization | 14,021 | 1.0 | 6,191 | 8.4 |
| Non-emergent hospitalization | 6,456 | 0.4 | 3,237 | 1.8 |
| No hospitalization | 239,585 | 16.3 | 283 | -10.9 |
| **81-85** |  |  |  |  |
| Emergent hospitalization | 15,629 | 1.1 | 7,077 | 10.8 |
| Non-emergent hospitalization | 5,002 | 0.3 | 3,958 | 1.8 |
| No hospitalization | 191,327 | 13.0 | 408 | -5.4 |
| **≥86** |  |  |  |  |
| Emergent hospitalization | 19,448 | 1.3 | 7,975 | 15.4 |
| Non-emergent hospitalization | 3,795 | 0.3 | 4,928 | 1.7 |
| No hospitalization | 169,833 | 11.6 | 649 | 0.2 |

Reference List

(1) Elixhauser A, Steiner C, Harris DR, Coffey RM. Comorbidity measures for use with administrative data. Med Care 1998 Jan;36(1):8-27.

(2) Klabunde CN, Potosky AL, Legler JM, Warren JL. Development of a comorbidity index using physician claims data. J Clin Epidemiol 2000 Dec;53(12):1258-67.

(3) Chitnis AS, Holzbauer SM, Belflower RM, Winston LG, Bamberg WM, Lyons C, et al. Epidemiology of community-associated Clostridium difficile infection, 2009 through 2011. JAMA Intern Med 2013 Jul 22;173(14):1359-67.

(4) Yun H, Kilgore ML, Curtis JR, Delzell E, Gary LC, Saag KG, et al. Identifying types of nursing facility stays using medicare claims data: an algorithm and validation. Health Serv Outcomes Res Method 2010 Jul 31;(10):100-10.

(5) Laffan AM, Bellantoni MF, Greenough WB, III, Zenilman JM. Burden of Clostridium difficile-associated diarrhea in a long-term care facility. J Am Geriatr Soc 2006 Jul;54(7):1068-73.
